# Differential Attraction of Malaria Mosquitoes to Volatile Blends Produced by Human Skin Bacteria

**DOI:** 10.1371/journal.pone.0015829

**Published:** 2010-12-30

**Authors:** Niels O. Verhulst, Rob Andriessen, Ulrike Groenhagen, Gabriella Bukovinszkiné Kiss, Stefan Schulz, Willem Takken, Joop J. A. van Loon, Gosse Schraa, Renate C. Smallegange

**Affiliations:** 1 Laboratory of Entomology, Wageningen University and Research Centre, Wageningen, The Netherlands; 2 Institut für Organische Chemie, Technische Universität Braunschweig, Braunschweig, Germany; 3 Laboratory of Microbiology, Wageningen University and Research Centre, Wageningen, The Netherlands; CNRS - Université Aix-Marseille, France

## Abstract

The malaria mosquito *Anopheles gambiae sensu stricto* is mainly guided by human odour components to find its blood host. Skin bacteria play an important role in the production of human body odour and when grown *in vitro*, skin bacteria produce volatiles that are attractive to *A. gambiae*. The role of single skin bacterial species in the production of volatiles that mediate the host-seeking behaviour of mosquitoes has remained largely unknown and is the subject of the present study. Headspace samples were taken to identify volatiles that mediate this behaviour. These volatiles could be used as mosquito attractants or repellents. Five commonly occurring species of skin bacteria were tested in an olfactometer for the production of volatiles that attract *A. gambiae*. Odour blends produced by some bacterial species were more attractive than blends produced by other species. In contrast to odours from the other bacterial species tested, odours produced by *Pseudomonas aeruginosa* were not attractive to *A. gambiae*. Headspace analysis of bacterial volatiles in combination with behavioural assays led to the identification of six compounds that elicited a behavioural effect in *A. gambiae*. Our results provide, to our knowledge, the first evidence for a role of selected bacterial species, common on the human skin, in determining the attractiveness of humans to malaria mosquitoes. This information will be used in the further development of a blend of semiochemicals for the manipulation of mosquito behaviour.

## Introduction

The African malaria mosquito *Anopheles gambiae* Giles *sensu stricto* (Diptera: Culicidae) (henceforth termed *A. gambiae*) is one of the most important malaria vectors because of its preference to feed on humans [1]. This mosquito species is nocturnal and is mainly guided by body odour to find its host [1]. Human body emanations contribute to the differential attractiveness of humans to anthropophilic mosquitoes and these differences remain relatively stable over time [2], [3], [4], [5], [6].

Several volatile compounds that mediate host-seeking behaviour in mosquitoes have already been identified and tested in laboratory, semi-field or field studies. Carbon dioxide (CO_2_), for example, plays an important role, presumably for mosquito activation and attraction over longer distances [7], [8], [9]. Ammonia, lactic acid and carboxylic acids are released from human skin and were attractive in laboratory, semi-field and field experiments when offered in a blend [10], [11], [12]. However, the factors determining the quantity of compounds and composition of the odour blend released from the human skin that mediate mosquito behaviour have received little attention.

Bacteria play an important role in the production of human odours [13]. Freshly secreted human sweat is odourless [14] and only limited attractive to *A. gambiae* compared to sweat incubated with skin bacteria [15]. In addition, there is a strong correlation between human body odour and the species composition of skin bacteria [16], [17], [18], [19]. Recent laboratory, semi-field, and field studies have shown that volatiles from bacteria obtained from human skin and grown *in vitro* on agar attract *A. gambiae* [20]. The identification of the volatiles present in the headspace of natural human skin bacterial cultures led to the development of a synthetic blend consisting of ten compounds that was attractive to *A. gambiae* [20].

Volatiles produced by bacteria are often widespread among bacterial taxa, but some transformations of organic compounds lead to the production of volatiles that are species- specific [21]. Bacillus subtilis has been associated with foot odour and corynebacteria species play a role in the synthesis of androsterone sulphate, which leads to a mixture of 16-androstenes, producing a typical axillary smell [22]. Staphylococci and corynebacteria play a role in the formation of volatile fatty acids [23], [24], which have a distinct sweaty odour. Of these two bacterial genera, only Staphylococcus species can convert branched-chain amino acids to highly odorous short-chain volatile fatty acids [24], that also play an important role in the host-seeking behaviour of A. gambiae [11], [12], [25].

Recent technological advances allow for high throughput 16s rDNA sequencing of the microbial community found on the human skin [26], [27], [28], [29]. Studies using this technique revealed that the distribution of bacterial species on the human skin depends on local skin site characteristics [28], [29] and that human individuals each have a unique composition of microbiota on their skin [27]. This composition remains relatively stable over time [28]. Many different species of bacteria have been identified, some of which had not previously been associated with human skin. Pseudomonads, for instance, are extremely versatile organisms, which are primarily found in soil, water, decomposing organic materials, and also found in the intestinal flora. They are not commonly associated with human skin [30], [31]. *Pseudomonas aeruginosa* is the best known *Pseudomonas* species on the human skin, often found as a secondary invader of wounds and regarded as a transient species [30]. A molecular study of the skin microbiota on the inner elbow, however, showed that *Pseudomonas* species were commonly present [31].

Volatiles produced by a mixture of human skin bacteria grown on agar attract *A. gambiae* [20]. However, the role of single skin bacterial species in the production of volatiles that mediate the host-seeking behaviour of mosquitoes has remained largely unknown. In the present study the relative attractiveness of five common skin bacterial species to *A. gambiae* was investigated. The species *Bacillus subtilis, Brevibacterium epidermidis, Corynebacterium minutissimum, Pseudomonas aeruginosa* and *Staphylococcus epidermidis* were chosen, because they are commonly found on the human skin and are associated with differences in body odour production [18], [31], [32], [33], [34], [35]. Headspace samples of batch cultures of each bacterial species were taken to identify potential attractive or repellent compounds.

## Results

### Response of *A. gambiae* to volatiles from different bacterial species

**Figure 1:**
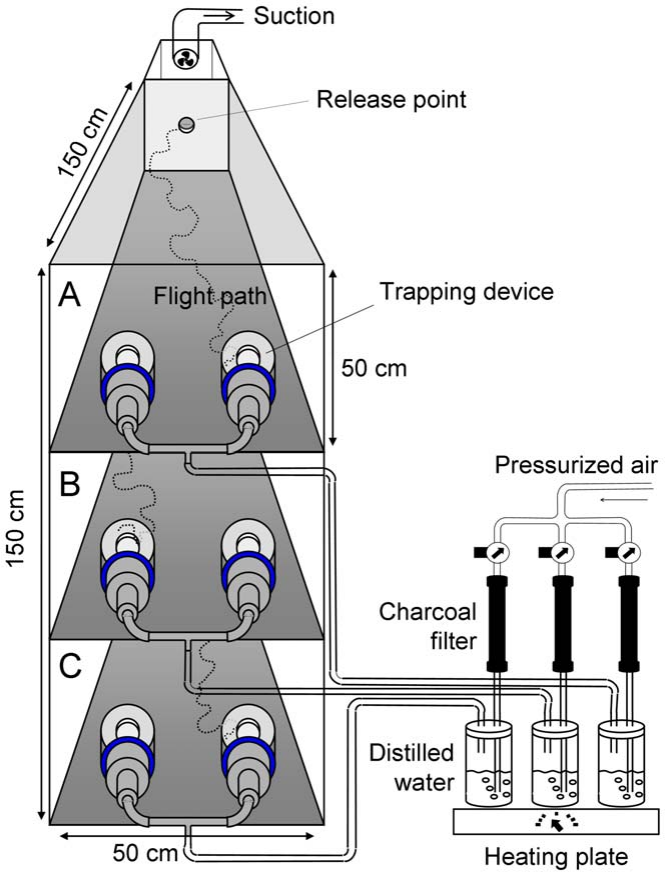
Schematic drawing of the dual-choice olfactometer used to examine the response of *Anopheles gambiae* to VOCs. The olfactometer contains three test chambers in a stacked configuration.

A dual-port olfactometer (Figure 1) [36] was used to evaluate host-seeking responses of female mosquitoes to volatile organic compounds (VOCs) produced by the bacterial species. The experiments with two unbaited traps showed no positional bias (χ^2^-test, d.f. = 1, P = 0.48). Trapping devices baited with standard medium caught similar numbers of mosquitoes as unbaited trapping devices (χ^2^-test, d.f. = 1, P = 0.56).

**Figure 2:**
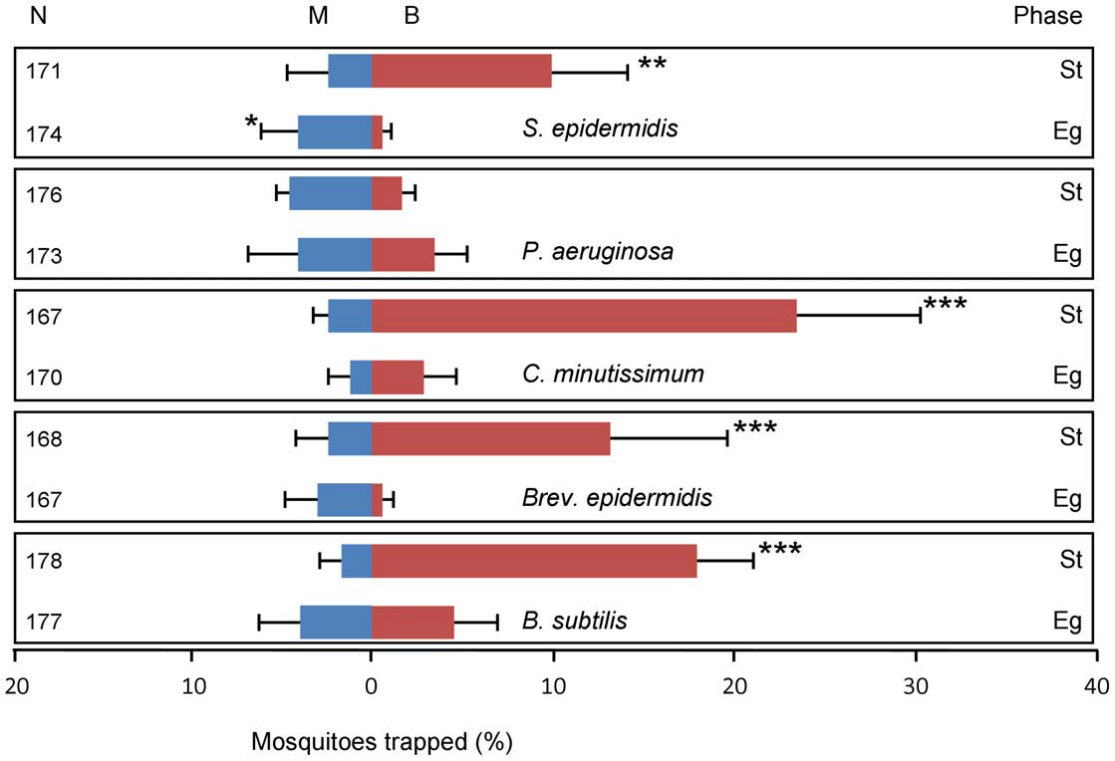
Response of *A. gambiae* to VOCs released from broths of five species of skin bacteria. Bacterial species were tested at the time of exponential growth (Eg) and during the stationary phase (St). The percentage of mosquitoes caught in the trapping device baited with the bacterial broth (B) and in the trapping device baited with medium alone (M) are given. N =  number of mosquitoes released. Error bars represent standard errors of the mean; ***: χ^2^-test P<0.001; **: χ^2^-test P<0.01: *: χ^2^-test P<0.05.

Bacterial broths of *B. subtilis, Brev. epidermidis, C. minutissimum, P. aeruginosa* and *S. epidermidis* (Table S1) were tested at the time of exponential growth and in the stationary phase, when the number of bacteria in the broth stayed constant (Figure S1). No preference of *A. gambiae* was found for trapping devices baited with bacteria at the time of exponential growth compared to trapping devices baited with standard medium alone (Figure 2; χ^2^-test, d.f. = 1, P>0.05), except for trapping devices baited with *S. epidermidis* which caught significantly fewer mosquitoes than medium alone (Figure 2; χ^2^-test, d.f. = 1, P = 0.034). Trapping devices with bacteria in the stationary phase caught significantly more mosquitoes than the trapping devices with clean medium for all species of bacteria (χ^2^-test, d.f. = 1, P≤0.005), except for *P. aeruginosa* (Figure 2; χ^2^-test, d.f. = 1, P = 0.13).

### Ranking different species of bacteria

**Figure 3:**
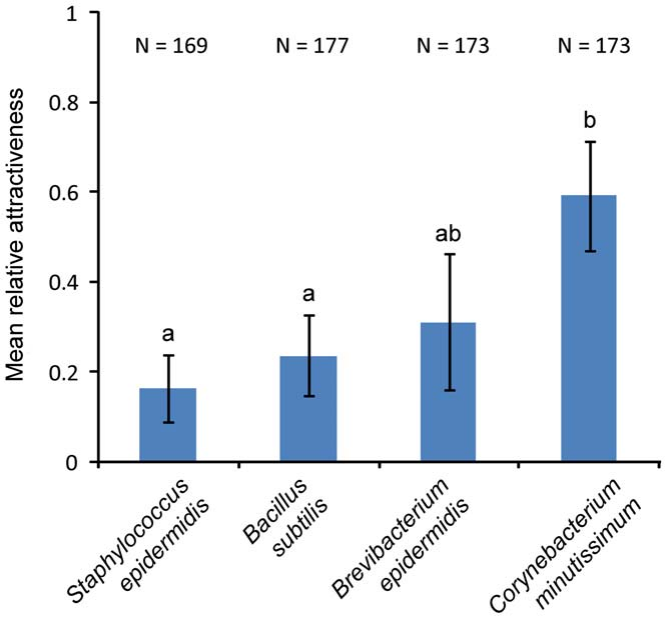
Ranking different species of skin bacteria for attractiveness to *A. gambiae.* Results of dual-choice olfactometer experiments in which VOCs of different skin bacterial species in liquid medium were tested for their attractiveness to *A. gambiae* against ammonia released from an LDPE sachet. Bars show the mean relative attractiveness (i.e. the number of mosquitoes entering the trapping device baited with bacteria as a proportion of the total number entering either this trapping device or the trapping device baited with ammonia). N = total number of mosquitoes released. Error bars represent standard errors of the mean. Means not sharing the same superscript letter differ significantly at P<0.05 (GLM).

In order to compare the response of *A. gambiae* to the different bacterial broths in the same series of experiments, broths of *B. subtilis, Brev. epidermidis, C. minutissimum,* and *S. epidermidis* in their stationary phase were tested against ammonia, which is a known attractant for *A. gambiae* [11], [37]. Olfactometer trapping devices baited with volatiles of *S. epidermidis, B. subtilis* and *Brev. epidermidis* caught fewer mosquitoes than trapping devices baited with ammonia, and thus, the relative attractiveness of these species was lower than 0.5 (Figure 3). The volatiles of *C. minutissimum* lured more mosquitoes into trapping devices than ammonia (Figure 3). Comparing the four species, the relative attractiveness of *C. minutissimum* was significantly higher than that of *S. epidermidis* and *B. subtilis* (Figure 3; GLM, d.f. = 3, P = 0.014 respectively P = 0.042). The relative attractiveness of *Brev. epidermidis* was equal to the relative attractiveness of the other bacterial species (GLM, d.f. = 4, P>0.05). No effects of bacterial density, temperature, humidity, air pressure, flight chamber, day and time of testing on the relative attractiveness were found (GLM, P>0.05) and these factors were, therefore, not included in the GLM model.

### Headspace analysis

More than 50 compounds were identified in the headspace of the standard medium. When the bacterial species were individually added to the medium and incubated, in total more than 150 volatile compounds were distinguished (Figure S2). As the bacterial broths of *B. subtilis, Brev. epidermidis, C. minutissimum* and *S. epidermidis* were attractive only in the stationary phase, we compared the headspaces of the stationary phase with those of the exponential growth phase. This comparison yielded ten headspace compounds (Table S2A, Figure S2) that were more abundant in broths in the stationary phase than in broths in the exponential growth phase. Comparing the headspace results of the *P. aeruginosa* broths in the stationary phase with the broths of the other bacterial species in the stationary phase resulted in four headspace compounds that were less abundant and one compound that was more abundant in *P. aeruginosa* broths than in broths of the other species (Table S2B, Figure S2). Butyl 2-methylbutanoate, pentathiane and 2-pentadecanone were more abundant in broths in the stationary phase than in broths in the exponential growth phase and were not found in the *Pseudomonas* broths. Butyl butyrate was the only compound found in higher amounts in the *P. aeruginosa* broth than in the broths of the other bacteria, except for the *S. epidermidis* broths (Table S2B, Figure S2).

The headspace samples of the individual bacterial species, *C. minutissimum, B. subtilis* and *Brev. epidermidis* contained many sulfur-containing compounds. *Staphylococcus epidermidis* headspace samples contained higher amounts of acetoin and 3-methyl-1-butanol compared to the headspace samples of the other bacterial species. The latter compound has been identified before in the headspace of *S. epidermidis* and other skin bacteria grown *in vitro* after isolation from human feet [20].

### Response of *A. gambiae* to volatiles identified in bacterial headspace

The addition of L-(+)-lactic acid (henceforth termed lactic acid) and carboxylic acids to ammonia had increased trap catches of *A. gambiae* in previous experiments [11], [12]. Therefore, we tested whether any of the above six compounds identified from the bacterial broth headspace samples could increase trap catches when added to a basic blend of ammonia, lactic acid and tetradecanoic acid [12].

**Figure 4:**
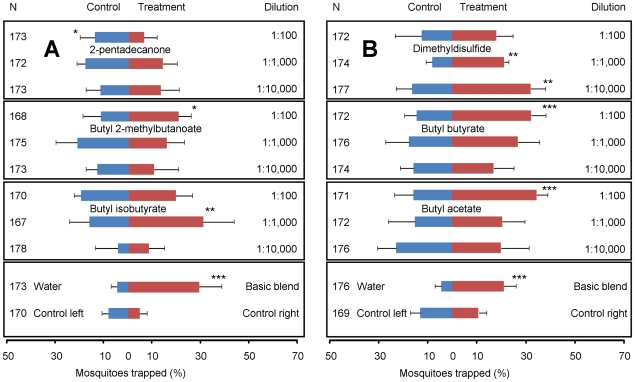
Responses of *A. gambiae* to individual compounds added to a basic blend in an olfactometer. Compounds were added in three concentrations (dilution 1∶100, 1∶1,000 or 1∶10,000) to the basic blend (ammonia, lactic acid, tetradecanoic acid) (basic blend + compound  =  Treatment) and tested against the basic blend (Control) alone in two series (A and B). N = number of mosquitoes released. Error bars represent standard errors of the mean; ***: χ^2^-test P<0.001; **: χ^2^-test P<0.01: *: χ^2^-test P<0.05.

Trapping devices baited with a basic blend of ammonia, lactic acid, and tetradecanoic acid in LDPE sachets caught significantly more mosquitoes than trapping devices baited with LDPE sachets containing water (Figure 4A,B; χ^2^-test, d.f. = 1, P<0.001), which is in accordance with the results of previous experiments [12].

Six of the 11 compounds selected for testing (Table S2) were commercially available and were examined for any impact on the response to the basic blend. Trapping devices baited with the basic blend and butyl 2-methylbutanoate, butyl butyrate or butyl acetate at the 1∶100 dilution caught significantly more mosquitoes than trapping devices baited with the basic blend alone (Figure 4A, B, χ^2^-test, d.f. = 1, P = 0.043, P<0.001 and P<0.001, respectively). By contrast, at the highest concentration tested (1∶100), 2-pentadecanone decreased the attractiveness of the basic blend significantly (χ^2^-test, d.f. = 1, P = 0.020). Butyl isobutyrate at a dilution of 1∶1,000 added to the basic blend caused a significantly increased response (χ^2^-test, d.f. = 1, P = 0.007). When dimethyldisulfide was added at the two lowest concentrations to the basic blend, trapping devices baited with this 4-compound-blend caught significantly more mosquitoes than trapping devices baited with the basic blend alone (Figure 4B, χ^2^-test, d.f. = 1, P = 0.001, and P = 0.003, respectively).

## Discussion

Four of the five bacterial species grown *in vitro* in liquid medium, produced volatiles that were attractive to the malaria mosquito *A. gambiae* at the concentrations tested (Figure 2). The ranking of these four bacterial species shows that some species commonly found on the human skin produce volatile blends that are more attractive to *A. gambiae* than volatile blends of other bacterial species. This shows that not all micro-organisms present on the human skin contribute equally to the attractiveness conferred to mosquitoes. *Corynebacterium minutissimum* ranked significantly higher than *B. subtilis* and *S. epidermidis* in the second series of experiments (Figure 3). Volatiles of *P. aeruginosa* were not attractive, and may even attenuate the effect of the volatiles from the other bacterial species.

The difference between the response of *A. gambiae* to volatiles released by skin bacteria in their exponential growth phase and stationary phase could in part be explained by the higher number of bacteria in the stationary phase. However, the headspace analyses show clearly that some compounds were present in the headspace samples of the bacterial broths in the stationary phase, but were not detected in samples of the bacterial broths in the exponential growth phase (Table S2A, Figure S2). This indicates qualitative differences in volatile production between the two growth phases.

The outermost layer of the human skin, the stratum corneum, is predominantly an oxic environment. However, certain micro-environments of the skin, such as glands and hair follicles, contain reduced levels of oxygen or no oxygen at all. Under these micro-aerophilic or anoxic conditions only bacteria with fermentative metabolic characteristics will be active [38]. During fermentation processes, organic compounds are converted into many different smaller compounds, often of a volatile nature [38]. *Staphylococcus epidermidis,* for example, metabolizes glycerol and lactic acid, forming large amounts of acetic and propionic acid as fermentation products [24]. Fermentation reactions, which occurred during the stationary phase of our experiments, could explain the high diversity of compounds found in the headspace of broths at this stage. Four of our bacterial species are known to express fermentation reactions in the absence of oxygen [23], [24], [39], [40]. Interestingly, *P. aeruginosa* grows best in the presence of oxygen or nitrate and has only limited fermentative capacities [41], [42]. To test whether fermentation in the absence of oxygen plays a role in the production of volatiles attractive to *A. gambiae*, the bacterial strains should be grown under anoxic conditions and tested for attractiveness to *A. gambiae*.

All five bacterial species could be grown in the same liquid medium and although this is probably not the most suitable medium for all species tested, it allowed for a comparison of the volatile blends produced against the same background. Trapping the volatiles from the headspace samples of bacteria using the CLSA technique resulted in more than 150 volatiles of which 11 were considered for further testing for their effect on *A. gambiae's* host-seeking behaviour (Table S2; Figure S2). Dimethyltetrasulfide, pentathiane, dimethylpentasulfide, hexathiepane and octasulfur were not commercially available. It is of interest to synthesize these compounds and test them for their attractiveness to *A. gambiae*. All of the remaining six compounds induced a behavioural response of *A. gambiae* in the olfactometer when tested in combination with the basic blend that consisted of ammonia, lactic acid and tetradecanoic acid (Figure 4A,B). Blends consisting of these nine compounds in varying ratios could further increase the mosquito catch and should be studied in future experiments under African semi-field or field conditions to reveal their potential use in mosquito control programmes. The chemically-driven analysis described in this study tends to reveal only highly abundant compounds, which are not necessarily the most behaviorally important ones. Bioassay-guided approaches that use a combination of gas chromatography and electrophysiological recording from olfactory organs (electroantennograpy) or single olfactory sensilla could reveal additional compounds occurring at low abundance that are therefore not detected by coupled gas chromatography – mass spectrometry, yet might exert behavioural activity. Ultimately, however, behavioural assays as applied in the current study are required to validate such activity. In addition, we note that the selection of compounds was not based on abundance as such but on the difference in abundance between exponential and stationary growth phases of the bacterial cultures and the absence of attraction by one of the bacterial species.

Based on the headspace composition it was not expected that butyl butyrate would increase the attractiveness of the basic blend to *A. gambiae*, since this compound was present in higher quantities in the headspace of *P. aeruginosa* broths than in that of broths containing *B. subtilis, Brev. epidermidis* or *C. minutissimum*. An explanation may be that the concentration of butyl butyrate tested in the olfactometer was different from the concentration released by *P. aeruginosa* broths, or other compounds present in the headspace samples of *P. aeruginosa* broths may have influenced the behavioural response of *A. gambiae*.

Dimethyldisulfide is the most interesting compound to test in semi-field and field experiments to evaluate as a mosquito bait under natural African conditions, because it was the only compound that increased the number of *A. gambiae* caught when added to the basic blend at the lowest concentration (Figure 4B). Dimethyldisulfide is a known bacterial volatile [43], [44], also found in human skin emanations [45] and has been shown to be attractive on its own and in blends with lactic acid and acetone in dual-port olfactometer experiments to the yellow fever mosquito *Aedes aegypti* L. [46], [47], [48].

We have shown previously that skin bacteria play an important role in the host-seeking behaviour of *A. gambiae* [20]. To find their host, *A. gambiae* females use host-specific cues in addition to CO_2_ and physical cues like heat and moisture [1]. As different bacterial species produce volatile blends of highly different composition it can be hypothesized that *A. gambiae* females are guided by volatiles released by skin bacteria that are specific for the human skin. The results from the present study support this hypothesis. *Pseudomonas aeruginosa* is present on the human skin [31], [49], but is also very common in the environment [50] and, therefore, not human-specific. This may explain why *A. gambiae* was not attracted to the volatile odour blend produced by this bacterial species (Figure 2). *Corynebacterium minutissimum* is strongly associated with human skin [51], [52], [53], and to our knowledge not commonly found on other substrates. It was also the most attractive bacterium in our study (Figure 3). In addition, corynebacteria and brevibacteria are present in high numbers on the human foot [38], which is the body part most attractive to *A. gambiae* [54].

Although not of human origin, bacterial volatiles also play an important role in the selection of oviposition sites by mosquitoes [55], [56], [57]. Comparing the volatiles released by human skin bacteria and bacteria obtained from oviposition sites can lead to the identification of new volatiles that attract or repel mosquitoes. If certain bacterial species produce compounds that attract both host-seeking mosquitoes and mosquitoes searching for an oviposition site, then odour-baited traps that attract mosquitoes in both physiological stages could be deployed to enhance the sensitivity of surveillance or for more effective mosquito control.

Skin micro-organisms are known to determine the human odour profile [16], [17], [18], [19]. In a previous study, using solid agar based medium [20], a mixture of human skin bacteria from the foot was found to produce compounds attractive to *A. gambiae*. In the present study it was shown that not all bacterial species produce blends of volatile organic compounds that attract *A. gambiae* and that some bacterial species produce odour blends that are more attractive than others. The GC-MS analyses of headspace volatiles led to the identification of compounds that, depending on the concentration tested, either increased or decreased the attractiveness of a basic blend. Attractive synthetic odour blends are of potential use in semiochemical-baited trapping systems for monitoring or controlling malaria vectors [58], [59], [60]. The present study provides further evidence that the composition of skin microbiota determines the attractiveness of humans to malaria mosquitoes. More detailed knowledge on the relationship between human skin microbiota and human body odour will contribute to our understanding of the evolutionary mechanisms underlying the anthropophilic host-seeking behaviour of mosquitoes and other blood-feeding insects and the development of novel means of vector-borne disease control [13].

## Materials and Methods

### Mosquitoes

The *Anopheles gambiae* Giles *sensu stricto* culture originated from Suakoko, Liberia and has been cultured in the laboratory in Wageningen since 1988. Mosquitoes were fed on human arms twice a week. During the present study, the *A. gambiae* colony was switched to membrane feeding, using human blood obtained from the blood bank (Sanquin Blood Supply Foundation, Nijmegen, The Netherlands). Blood was offered through Parafilm® using a Hemotec® PS5 (Discovery Workshops, UK) feeder at 38°C. During feeding, a sock releasing human odour was wrapped around the membrane and 5% CO_2_ (human equivalent, [9]) was added next to the membrane at 250 ml/min. Olfactometer experiments showed no behavioural differences between mosquitoes originating from the colony reared on human arms and mosquitoes originating from the colony reared on membranes (G. Bukovinszkiné Kiss, unpublished data). Mosquitoes originating from the colony reared on membranes were used during the olfactometer experiments testing synthetic compounds.

Adult mosquitoes were maintained in 30-cm cubic gauze-covered cages in a climate-controlled chamber (27±1°C, 80±5% RH, LD 12∶12). They had access to a 6% (w/v) glucose solution on filter paper. Eggs were laid on wet filter paper and placed in tap water in plastic trays. Larvae were fed daily with Tetramin® baby fish food (Melle, Germany). Pupae were collected daily and placed in 30-cm cubic cages for eclosion.

### Bacteria

Five bacterial species (*Bacillus subtilis, Brevibacterium epidermidis, Corynebacterium minutissimum, Pseudomonas aeruginosa, Staphylococcus epidermidis*) commonly found on human skin were purchased from Deutsche Sammlung von Mikroorganismen und Zellkulturen GmbH (DSMZ, Braunschweig, Germany) or from the Laboratory of Microbiology (Wageningen University and Research Centre, Wageningen, The Netherlands) (Table S1). All species were initially cultivated in species-specific media to enable exponential growth (Table S3). Subsequently, each species was incubated in a standard liquid medium (Table S4) to exclude the possible effect of different media on volatile production and thereby mosquito responses. Bacteria were incubated in tubes containing 5 ml standard medium at 34°C shaking at 225 rpm (Innova TM 4000, New Brunswick Scientific Co.). After incubation, bacteria were stored in glycerol stocks containing 300 µl glycerol and 700 µl medium at −80°C.

To assess the number of bacteria tested in each experiment, reference graphs were created to determine the correlation between the number of colony-forming units (CFUs) in the standard medium and the optical density of the medium, measured by a spectrophotometer (SmartSpec^tm^ 3000, Bio-Rad). First, the optical density of at least six samples of 1 ml during growth of the bacteria was measured at 620 nm. Bacterial concentrations resulting in an extinction value measured with the spectrophotometer above 1 were diluted with standard medium, because the spectrophotometer is not accurate at optical densities above this value.

Secondly, for quantification of the bacterial population, each sample that had been measured in the spectrophotometer was diluted decimally, spread on species-specific agar (Table S3) plates and incubated at 34°C. CFUs were counted on dilution plates (between 25 and 250 colonies per plate), 3–6 days after spread plating. Count data were converted to number of bacteria per millilitre of liquid medium (determined by number of CFUs). The correlation between optical density and number of bacteria was fitted with a linear regression line, which was used as a reference line for calculation of the bacterial numbers tested in each experiment (Microsoft Excel; Table S5; example for *C. minutissimum* given in Figure S1A).

Next, for each bacterial species the optical density of the broth was measured at regular intervals to determine the growth curve. The growth curve of bacteria in a batch culture follows an S-curve [61]. In the exponential growth phase, which follows upon a lag phase, the bacterial growth rate will reach its maximum. This stage is followed by the stationary phase, in which the growth rate slows down due to nutrient depletion and/or accumulation of toxic products, and in which the maximum number of bacteria is reached. The exponential growth and stationary phase of each bacterial species were determined by fitting the observed optical density values to a logistic S-shaped curve (Genstat, release 12.1; Table S6; Figure S1B). For visualization, the growth curve describing the number of bacteria over time was plotted by integrating the reference line and fitted S-shaped curve (Figure S1C, S3).

### Olfactometer bioassays

A triple cage dual-port olfactometer (Figure 1, Tupola, The Netherlands) [36] was used to evaluate host-seeking responses of female mosquitoes to volatile organic compounds (VOCs) produced by the bacterial species. Pressurized air was charcoal-filtered, humidified, and passed through two poly-methyl-methyl-acrylaat (PMMA) mosquito trapping devices equipped with funnels [62], which were linked to both ports (diameter 5 cm, 25 cm apart) of the olfactometer. The air entered the flight chamber (polycarbonate,1.50×0.50×0.50 m) at a rate of 0.21±0.02 m/s. Temperature, humidity and air pressure were recorded using dataloggers (MSR145S, MSR Electronics GmBHm, Switzerland). The air entered the two trapping devices at a temperature of 27.0±1.0°C, and had a relative humidity above 80%. The air temperature in the flight chamber was 26.3±0.9°C and its relative humidity was 66.0±8.2%. The experimental room was maintained at a temperature of 26.3±1.0°C and a relative humidity of 59.6±8.3%.

Experiments were prepared and performed according to the methods described by Smallegange *et al.* [11]. For each test 30 (mated) female mosquitoes of 5–8 d old, which had never been offered a blood meal, were selected 14–18 h before the experiment and placed in a cylindrical release cage (d = 8 cm, h = 10 cm) with access to tap water from damp cotton wool. The experiments were performed during the last 4 h of the scotophase, when *A. gambiae* females are known to be highly responsive to host odours [63], [64].

In each trial, test odours were released in the air stream before a group of mosquitoes was set free from a cage which was placed at the downwind end of the flight chamber, 1.50 m from the two ports. Mosquitoes were left in the flight chamber for 15 min. Specimens that entered each of the two trapping devices were counted at the end of the experiments after anesthesia with CO_2_. Mosquitoes remaining in the flight chamber were removed with a vacuum cleaner. Each trial started with a fresh batch of mosquitoes, clean trapping devices, and new stimuli.

The sequence of test odours was randomized on the same day, between days and between the three layers of the olfactometers. Each treatment was repeated six times and test stimuli were alternated between right and left ports between different replicates to rule out any positional effects. Surgical gloves were worn by the researcher at all times to avoid contamination of equipment with human volatiles.

### Response of *A. gambiae* to volatiles from different species of bacteria

The volatiles of a broth of each bacterial species (Table S1) in liquid medium (Table S4) were tested for their effect on *A. gambiae*. Broths were tested at the time of exponential growth and in the stationary phase, when the number of bacteria in the broth stayed constant. Broths were incubated on the day before the experiments and kept at 4°C overnight. Before the experiments, broths were incubated one more hour at 34°C, 225 rpm.

Five minutes before each experiment, 100 µl of the bacterial broth was spread on a sandblasted glass slide (75×25 mm) and positioned in the middle of one of the trapping devices of the olfactometer. Clean standard medium (100 µl) was used as a control and placed on a sandblasted glass slide in the other trapping device. The response of *A. gambiae* to the medium itself was tested in a separate experiment. One hundred µl of medium on a sand-blasted glass slide was placed inside one of the trapping devices in the olfactometer and tested against an unbaited trapping device. Two unbaited traps were offered to test the response of the mosquitoes when no odour was present and to test the symmetry of the system.

### Ranking different species of bacteria based on mosquito response

In order to compare the responses of *A. gambiae* to the different bacterial broths, broths of *B. subtilis, Brev. epidermidis, C. minutissimum,* and *S. epidermidis* were tested against ammonia, which is a known attractant for *A. gambiae* [11], [37]. Because all bacterial species were tested in the same randomized series of experiments and against a standard of ammonia, it was possible to rank the bacteria according to their relative attractiveness [5].

A volume of 100 µl of the bacterial broths on a sand blasted glass slide was tested against ammonia (100 µl, 25% in water; analytical grade, Merck) released from Low Density PolyEthylene (LDPE; Audion Elektro, The Netherlands) sachets (25×25 mm, 0.20 mm thick) [65]. LDPE sachets loaded with ammonia were suspended from a hook inside the olfactometer trapping device (Figure S4).

### Headspace volatile analysis

Bacterial volatiles were trapped on active charcoal using a Closed-Loop Stripping Apparatus (CLSA) [66], [67]. In this system, air is continuously pumped (MB-21E, Senior Flextronics, USA) through a closed system over the bacterial broth present in a closed 250 ml Erlenmeyer flask and through an activated charcoal filter (5 mg, Chrom Tech, Germany), in which the volatiles were absorbed.

The Erlenmeyer flask was first rinsed with acetone (Technical grade, CVH Chemie-Vertrieb GmbH & Co, Germany), next with distilled water and autoclaved before use. The flask was filled with 50 ml standard medium (Table S4) and 200 µl bacterial suspension from a glycerol stock was added. Next, the broth was placed in an incubator at 34°C. After 3 h the broth was placed for 3 h (exponential growth phase) or 20 h (stationary phase) at 34°C in the CLSA setup. The airstream in the CLSA setup was directed towards the surface of the incubation mixture, leading to constant mixing of the culture. Trapped volatiles were extracted from the charcoal filter by rinsing the filter three times with 10–15 µl dichloromethane (≥99.8%, Merck, Germany). Before use, the active charcoal filter had been rinsed with pentane (≥99%, Sigma-Aldrich, Germany), methanol (≥99.8%, Merck, Germany), and dichloromethane (≥99.8%, Merck, Germany).

Extracts were analyzed by gas chromatography – mass spectroscopy (GC-MS, GC 7890A/MSD 5975C, Agilent Technologies, USA). The GC-MS system was equipped with a split/splitless injector and an MS fused silica capillary column HP-5 MS (30 m, 0.25 mm internal diameter, 0.25 microns phase thickness, Agilent Technologies, USA) with He as the carrier gas (1.2 ml/min). The GC oven temperature was kept at 50°C for 5 min, followed by raising the temperature with 5°C/min to 320°C. Mass-spectra were recorded by electron impact ionization at 70 eV.

Compounds were identified by comparison of their mass spectra and gas chromatographic retention indices with those of authentic reference compounds and use of commercial mass spectral libraries (Wiley 7, NIST 08). Quantification of abundance was done in a semi-quantitative way by assigning each component to one of four classes dependent on the relative abundance in the total ion chromatogram 0 – 0.5%, 0.5 – 10%, 10 – 30%, 30 – 100%, relative to the total ion count of the largest peak in the chromatogram.

Two criteria were used for the selection of headspace compounds that were potentially attractive or repellent to *A. gambiae*. 1) The abundance of compounds in the stationary phase of broths of *B. subtilis, Brev. epidermidis, C. minutissimum* and *S. epidermidis* that were attractive to *A. gambiae* was compared with the abundance of compounds in the exponential growth phase of these broths, which were unattractive to *A. gambiae*. Compounds with a higher overall abundance in the stationary phase or a higher overall abundance in the exponential growth phase were selected. 2) The abundance of compounds in the stationary phase of broths of *B. subtilis, Brev. epidermidis, C. minutissimum* and *S. epidermidis*, which were attractive to *A. gambiae*, were compared with the abundance of compounds in the stationary phase of *P. aeruginosa* broths. The latter did not attract *A. gambiae*. Compounds with a higher overall abundance in *P. aeruginosa* broths or a higher overall abundance in the broths of the other bacterial species were selected.

### Response of *A. gambiae* to volatiles identified in bacterial headspace samples

Six volatile compounds originating from bacterial broths and identified by GC-MS (butyl acetate, butyl isobutyrate, butyl butyrate, butyl 2-methylbutanoate, dimethyldisulfide and 2-pentadecanone) were commercially available and tested in the olfactometer. All compounds were purchased from Sigma Aldrich (Germany) with purity levels ranging between 97 and 99%. Each compound was tested in three dilutions in paraffin oil (Merck, 1∶100; 1∶1,000 and 1∶10,000) and released from LDPE sachets (25×25 mm, 0.20 mm thick) [65]. Each sachet contained 100 µl of the dilution tested.

It was tested whether any of the above six compounds identified from the bacterial broth headspace samples could increase trap catches when added to a basic blend of ammonia, L-(+)-lactic acid and tetradecanoic acid [12]. Ammonia (25% in water; analytical grade, Merck) and tetradecanoic acid (>99%, Sigma) were released from LDPE sachets 0.03 mm thick, while lactic acid (88–92% aqueous solution, Riedel-de Haën) was released from LDPE sachets of 0.05 mm thick. These LDPE thicknesses were chosen based on the results of previous experiments (R.C. Smallegange, unpublished data). Sachets were 25×25 mm in size and contained either 100 µl ammonia solution, 100 µl lactic acid solution, or 50 mg tetradecanoic acid.

To test whether the six selected compounds identified from bacterial broth headspace samples could increase trap catches of *A. gambiae* when added to the basic blend, each dilution of the compounds was offered in an olfactometer trapping device together with the basic blend and tested against a control of a trapping device that contained only the basic blend and an LDPE sachet with paraffin oil. The six compounds were tested in two series (A and B) of three compounds. Each dilution of the compounds was tested six times and the position of the two odour sources was alternated between right and left ports among the replicate experiments. To test the symmetry of the system, two unbaited traps were offered. In addition, the basic blend was tested against LDPE sachets of the same size and thickness filled with distilled water to show that this blend is attractive.

### Statistics

The spectrophotometer extinction values measured at different bacterial densities were fitted to a linear regression line (Microsoft Excel). To plot growth curves of each bacterial species, the extinction values measured at different incubation times during growth of each bacterial species were fitted to a logistic S-shaped curve (Genstat, release 12.1).

For the two-choice test in the olfactometer a χ^2^-test was used to analyze whether the total (i.e. sum of all replicates) number of mosquitoes that was trapped in the treatment trapping device and the total number that was trapped in the control trapping device differed from a 1∶1 distribution (P<0.05).

The relative attractiveness of each bacterial species was tested with a Generalized Linear Model (GLM; Genstat, release 12.1; Binomial, linked in logit, dispersion estimated). The relative attractiveness is expressed as the number of mosquitoes caught in the trapping device baited with the bacterial odour divided by the total number of mosquitoes trapped in both trapping devices (bacteria trap + ammonia trap) during each experiment [5], [68]. Two-sided t-probabilities were calculated to test pairwise differences between means. Effects were considered to be significant at P<0.05. For each series of experiments the effects of bacterial density, temperature and humidity of the flight chamber, air pressure, flight chamber (Figure 1: A, B, C), day and time of testing on the relative attractiveness were tested and fitted as parameters in the GLM model when significant.

## Supporting Information

Figure S1 **Combining spectrophotometer extinction values and bacterial numbers to plot a growth curve.** *Corynebacterium minutissimum* is shown as an example. A: Correlation between the number of bacteria (determined by colony forming units, CFU) in standard medium and the optical density (extinction) of the medium in a spectrophotometer. B: Growth over time of *C. minutissimum* measured as optical density (extinction) and fitted by a logistic S‐shaped curve (Genstat, release 12.1). C: Combining data from graphs A and B in a growth curve represented by the number of *C. minutissimum* (CFU) in standard medium over time.
(TIF)

Figure S2 **Selection of GC‐MS chromatograms of growth medium and bacterial headspace.** A) Medium, B) *C. minutissimum*, C) *B. subtilis*, D) *S. epidermidis*, E) *B. epidermidis*, F) *P. aeruginosa.*
(PDF)

Figure S3 **Growth curve of the five bacterial species in standard liquid medium.** Bacterial numbers were determined by counting colony forming units (CFUs).
(TIF)

Figure S4 **LDPE sachets, used to test synthetic compounds, were suspended from a hook inside an olfactometer trapping device.** Gauze cover of trapping device not shown.
(TIF)

Table S1 **Bacterial species used for experiments and headspace analyses.**
(DOC)

Table S2 **Relative abundance of compounds that were potentially attractive or repellent to *A. gambiae*.**
(DOC)

Table S3 **Medium ingredients used for initial growth of each bacterial species (DSMZ, Germany).**
(DOC)

Table S4 **Ingredients of standard liquid medium used for growth of the five bacterial species.**
(DOC)

Table S5 **Correlation between the number of CFUs in liquid medium and the extinction coefficient.**
(DOC)

Table S6 **Exponential growth rate and fitted equation of the bacterial species in liquid medium.**
(DOC)
