# Supplementary figures and images for: Differential Attraction of Malaria Mosquitoes to Volatile Blends Produced by Human Skin Bacteria
Source: PLoS One. 2010 Dec 30;5(12):e15829. doi: 10.1371/journal.pone.0015829 (PMC3012726; doi:10.1371/journal.pone.0015829)

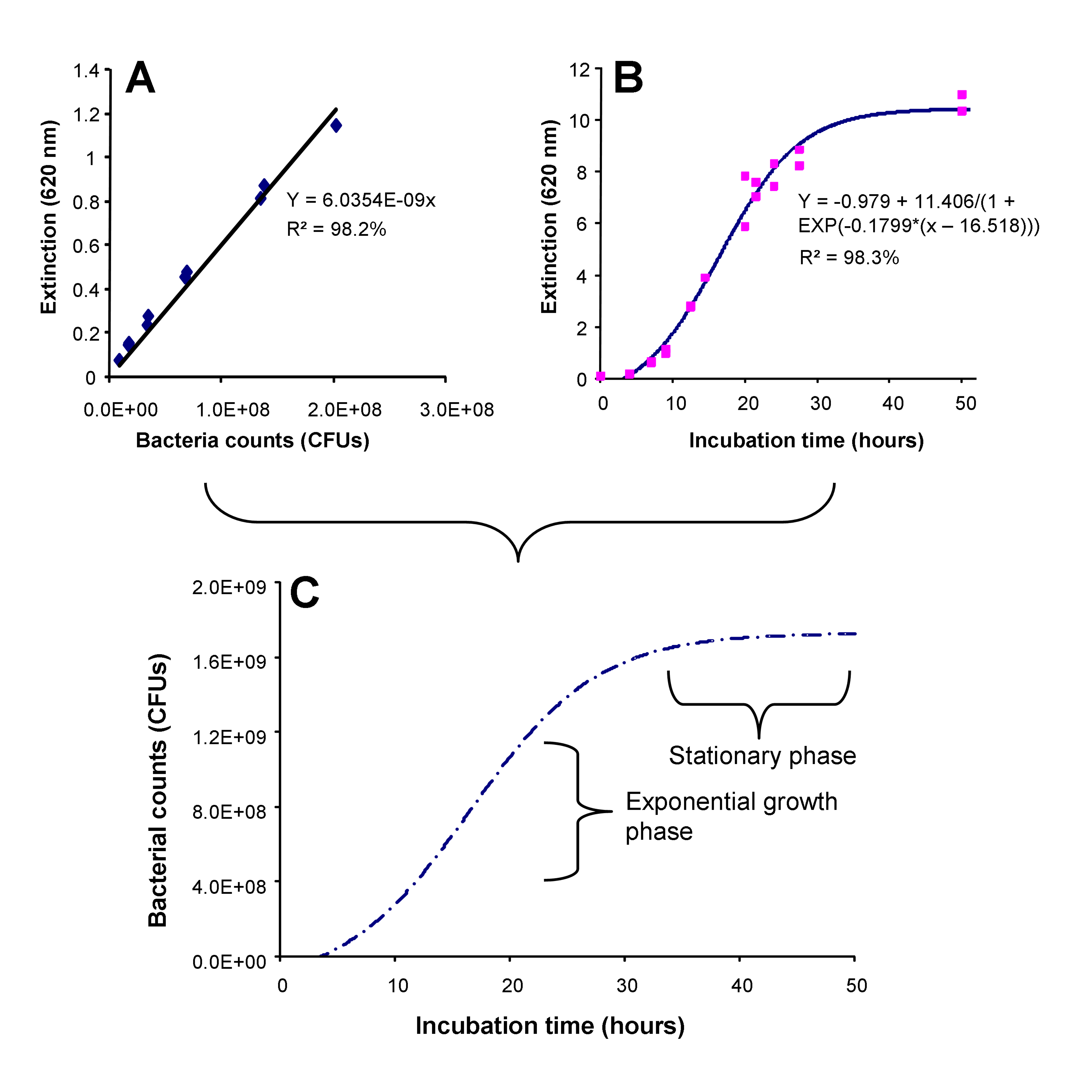

Supplement: Figure S1 — Combining spectrophotometer extinction values and bacterial numbers to plot a growth curve. Corynebacterium minutissimum is shown as an example. A: Correlation between the number of bacteria (determined by colony forming units, CFU) in standard medium and the optical density (extinction) of the medium in a spectrophotometer. B: Growth over time of C. minutissimum measured as optical density (extinction) and fitted by a logistic S‐shaped curve (Genstat, release 12.1). C: Combining data from graphs A and B in a growth curve represented by the number of C. minutissimum (CFU) in standard medium over time. (TIF) [file pone.0015829.s001.tif]

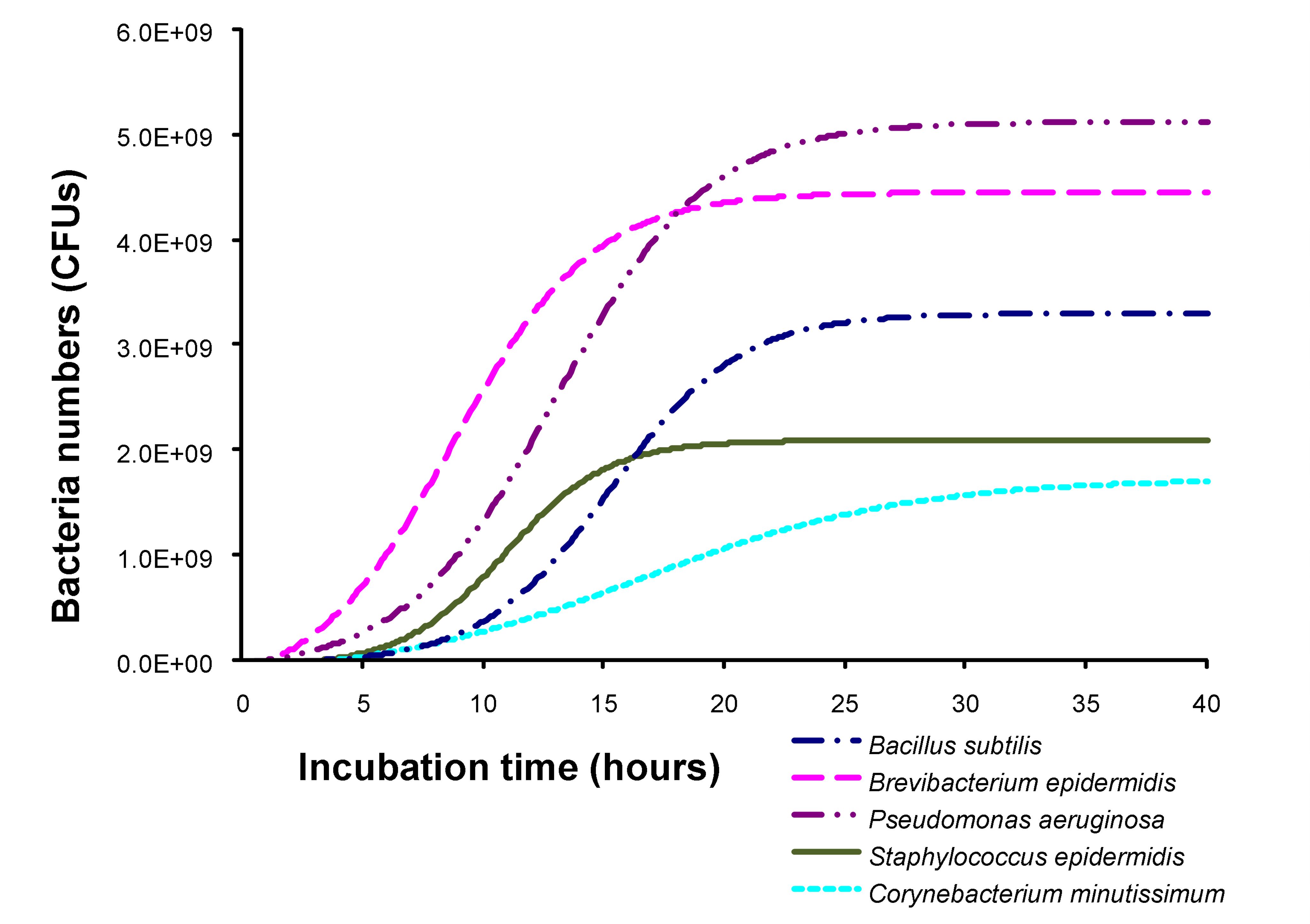

Supplement: Figure S3 — Growth curve of the five bacterial species in standard liquid medium. Bacterial numbers were determined by counting colony forming units (CFUs). (TIF) [file pone.0015829.s003.tif]

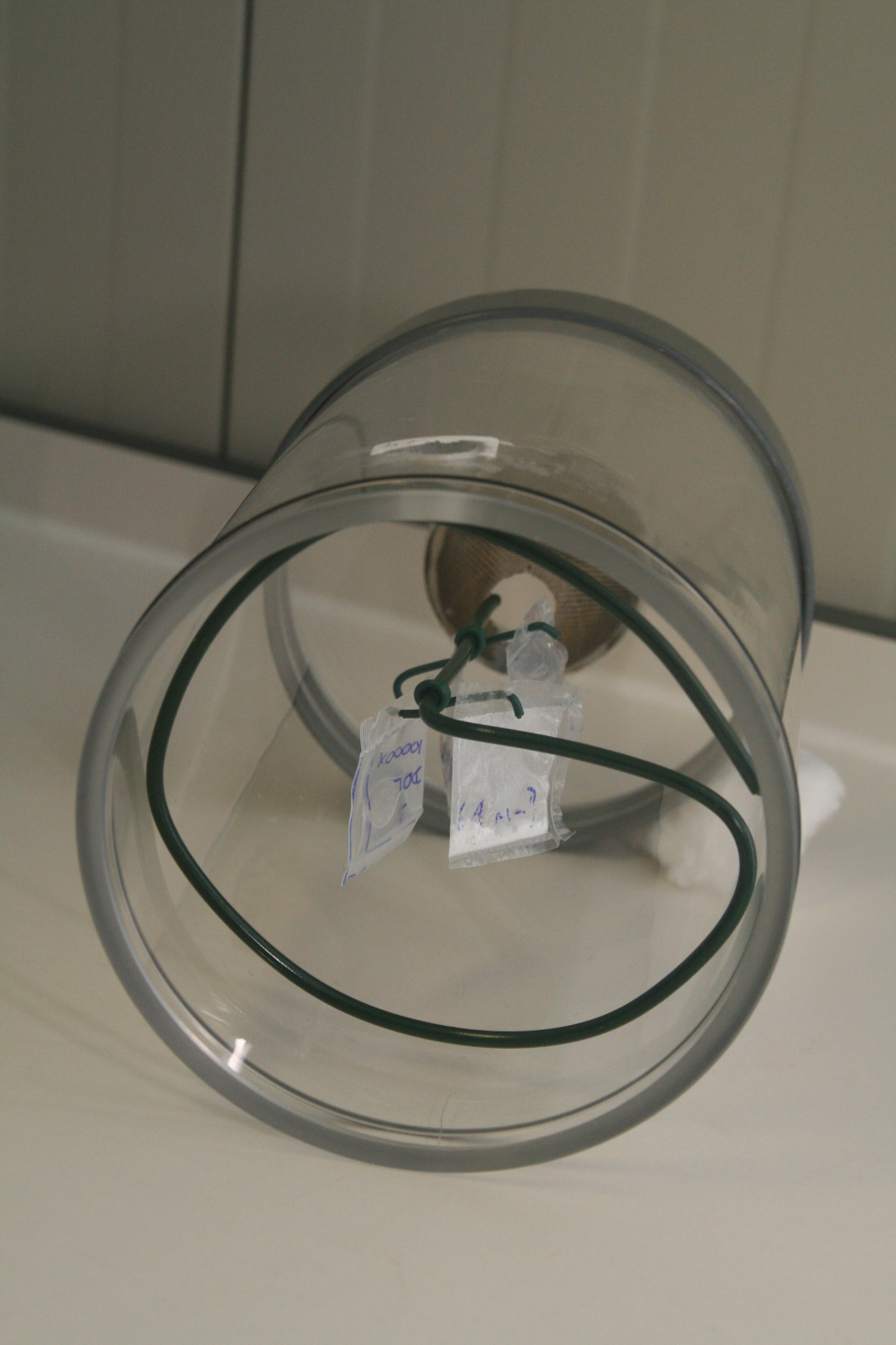

Supplement: Figure S4 — LDPE sachets, used to test synthetic compounds, were suspended from a hook inside an olfactometer trapping device. Gauze cover of trapping device not shown. (TIF) [file pone.0015829.s004.tif]
