# Supplementary material for: Differential Attraction of Malaria Mosquitoes to Volatile Blends Produced by Human Skin Bacteria
Source: PLoS One. 2010 Dec 30;5(12):e15829. doi: 10.1371/journal.pone.0015829 (PMC3012726; doi:10.1371/journal.pone.0015829)
Supplement: Figure S2 — Selection of GC‐MS chromatograms of growth medium and bacterial headspace. A) Medium, B) C. minutissimum, C) B. subtilis, D) S. epidermidis, E) B. epidermidis, F) P. aeruginosa. (PDF) [file pone.0015829.s002.pdf]

Figure S2 GC-MS chromatograms of medium and bacterial headspace

Figure S2A Total ion chromatogram of medium headspace and compounds identified. Largest peaks are indicated with a letter which corresponds with the list of compounds below.

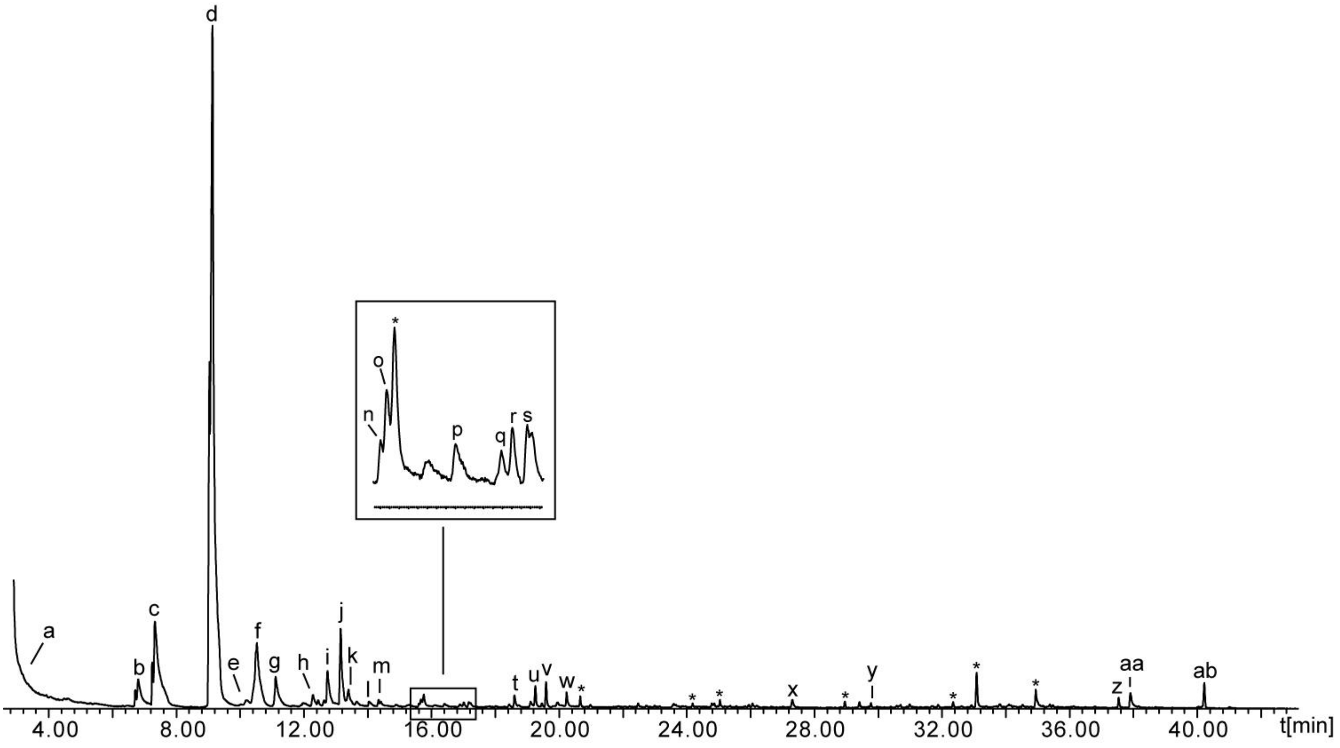

GC[a)]	Compound	Retention index[b)]	Intensity
a	1-Butanol	n.d.[c)]	++++
b	Cyclohexanone	901	++
c	2,5-Dimethylpyrazine	915	+++
d	Benzaldehyde	963	++++
e	Butylbutyrate	997	++
f	Trimethylpyrazine	999	+++
g	2-Acetylthiazol	1016	++
	2-Hydroxybenzaldehyde	1040	+
h	Pyrazin M: 136	1050	++
	m/z: 57, 85, 41, 129	1055	+
	m/z: 57, 85, 41, 129	1060	+
i	Acetophenone	1063	++
j	2-Ethyl-3,6-dimethylpyrazine	1075	++
k	Pyrazin M: 136	1082	++
l	Nonanal	1104	++
m	m/z: 43, 123, 101, 138	1111	+
	m/z: 43, 69, 131, 114, 85	1113	+
n	Pyrazin M: 164	1151	++
o	2,6-Di-(1-Methylethyl)pyrazin	1153	++
*	Silanoxan	1156	++

p	Naphthalene	1176	++
q	Pyrazin, M=164	1193	++
r	Dimethyl-(2-methylpropyl)-pyrazine M=164	1196	+
s	Decanal	1201	+
	m/z: 107, 135, 91, 150, 80	1203	+
t	2-Butyl-3,6-dimethylpyrazine	1253	++
	α-Ethylidenebenzeneacetaldehyde	1272	++
u	m/z: 145, 76, 120, 46, 192	1277	++
	Isobornylacetate	1285	++
v	m/z: 145, 118, 192, 46	1289	++
	Pyrazine	1302	++
w	2,5-Dimethyl-3-(3-methylbutyl)-pyrazine	1313	++
*	Siloxane*	1329	++
*	Unknown	1465	++
	Hexasulfur	1487	++
	Pentadecane	1498	+
*	Cyclosiloxane*	1499	+
	sesquiterpene	1513	+
	m/z: 71, 57, 85, 43, 99	1537	+
	m/z: 205, 190, 174, 73	1542	+
x	Hexadecane	1596	++
*	Cyclosiloxane	1669	+
y	Hydrocarbon	1706	+
*	Unknown	1829	+
	m/z: 157, 143, 185, 270	1857	+
*	Bis(2-methylpropyl)phthalat	1866	++
	m/z: 205, 217, 220, 175	1917	+
	m/z: 160, 145, 270, 95, 105	1943	+
*	Dibutylphthalat	1961	++
	m/z: 239, 183, 253, 268	1988	+
z	2-Ethylhexyl dodecanoate,*	2099	++
aa	m/z: 121, 179, 137, 292	2120	++
ab	m/z: 149, 91, 121, 79	2253	++
	m/z: 112, 70, 57	2300	+

[a)]Peak in chromatogram, [b)]Abundance relative to the largest peak in the total ion chromatogram: + 0 – 0.5%; ++ 0.5 – 10%; +++ 10 – 30%; ++++ 30 – 100%, [c)]not determined, *) artifact

Figure S2B Total ion chromatogram and compounds identified of *C. minutissimum* headspace.

Largest peaks are indicated with a letter which corresponds with the list of compounds below.

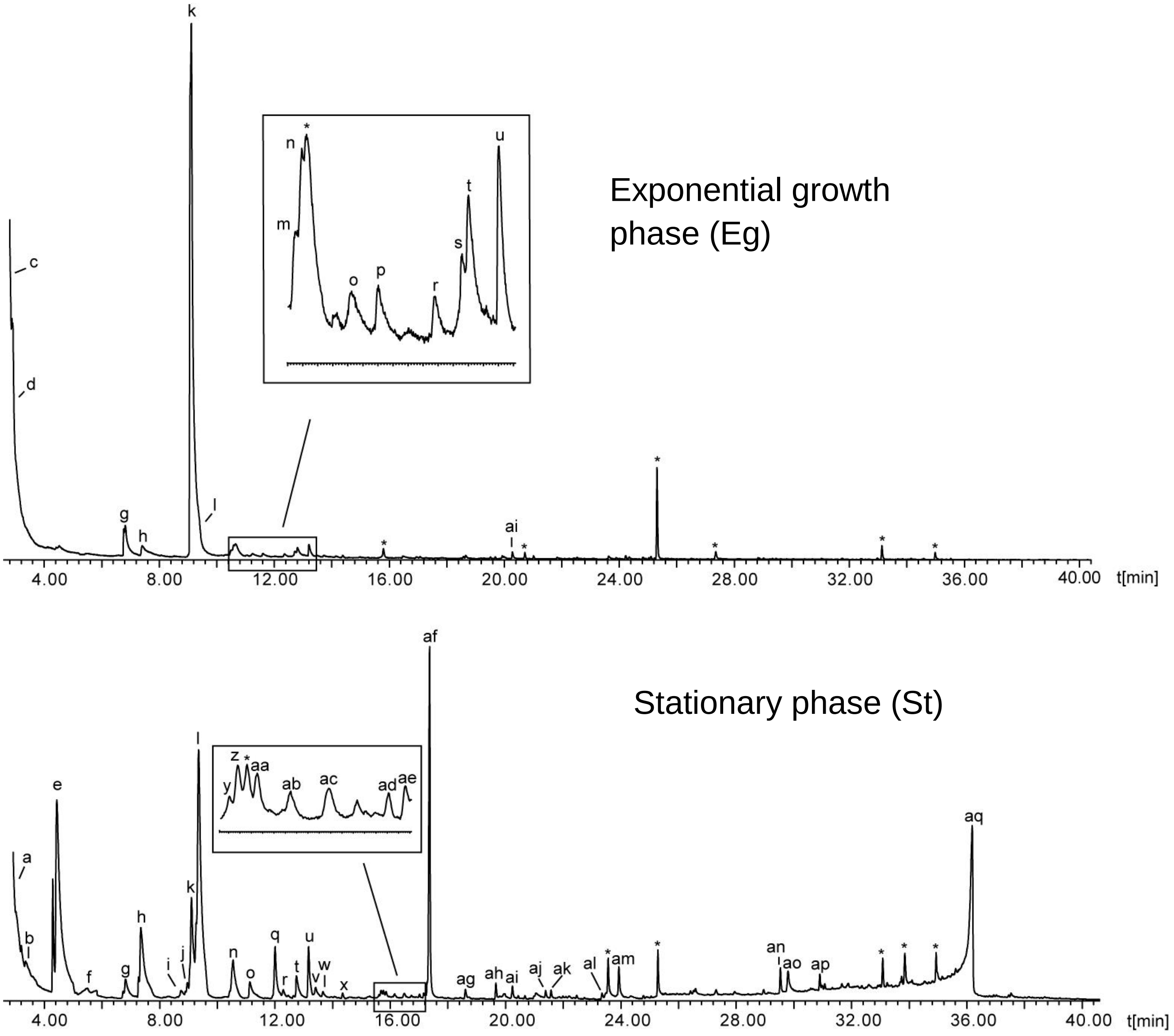

GC[a)]	Compound	Retention index[b)]	Intensity Eg	Intensity St
a	Ethylacetate*	n.d.[c)]		++++
b	Toluol	n.d.		++
c	1-Butanol	n.d.	++	
d	3-Methyl-1-butanol	n.d.	++	
e	Butylacetate	826		++++
f	3-Methylbutanoic acid	863		++
g	Cyclohexanone	901	++	++
h	2,5-Dimethylpyrazine	915	++	++++
i	3-Hydroxy-2-heptanon or isomeric	952		++
j	Butylisobutyrate	958		++

k	Benzaldehyde	963	++++	++++
l	Dimethyltrisulfide	968	++	++++
m	Butylbutyrate	997	+	
n	Trimethylpyrazine	999	++	+++
o	2-Acetylthiazol	1016	+	++
p	2-Ethyl-1-hexanol*	1029	+	
q	Butyl-2-methylbutanoate	1042		+++
r	Pyrazine, M: 136	1050	+	++
s	*S*-Methylmethanthiosulphonate	1062	+	
t	Acetophenone	1063	++	++
u	2-Ethyl-3,6-dimethylpyrazine	1075	++	+++
v	Pyrazine, M: 136	1082		++
w	m/z: 123, 58, 43, 138	1090		++
x	m/z: 43, 123, 101, 138	1111	+	++
y	2,6-Di(1-Methylethyl)pyrazine	1153		++
z	Pyrazine M: 150	1155	+	++
*	Silanoxane	1156	++	
aa	7-Methyl-2-nonanone	1160		++
ab	Methyl(2-methyl-3-furyl)disulfide	1168		+
ac	m/z: 160, 91, 45	1179		+
	Pyrazine, M=164	1193	+	
ad	Dimethyl-(2-methylpropyl)-pyrazine, M=164	1196	+	
ae	Decanal	1201		++
af	Dimethyltetrasulfide	1208		++++
ag	2-Butyl-3,6-dimethylpyrazine	1253	+	++
	Isobornylacetate	1285	+	
ah	2-Undecanone	1292		++
	m/z: 57, 143, 87, 69, 159	1301	+	
	Pyrazine	1302	+	+
ai	2,5-Dimethyl-3-(3-methylbutyl)-pyrazine	1313	+	++
	m/z: 69, 43, 85, 111, 154	1321		+
	m/z: 138, 95, 83, 193, 57	1340	+	
aj	10-Methyl-2-undecanone	1359		+
ak	9-Methyl-2-undecanone	1363		++
	Artifact	1371	+	
	Tetradecane	1397	+	+
al	Isobutyl-2-phenylacetate	1432		++
	Pentathiane	1437		++
am	Dimethylpentasulfide	1455		++
	Artifact	1465	+	
	Artifact	1470	+	
	Hexasulfur	1487	+	
	5-Methyl-2-phenyl-2-hexenal	1488	+	+
	12-Methyl-2-tridecanone	1557		+
	11-Methyl-2-tridecanone	1567		++
	Hexadecane	1596	+	++
	Lenthionine	1623		+
	Cyclosiloxane	1669		++
an	2-Pentadecanone	1695		++
ao	Hexathiepane	1707		++

ap	14-Methyl-2-pentadecanone	1760		++
	Octadecane	1796		++
	m/z: 253, 268, 185, 171, 85	1800	+	
	m/z: 229, 145, 159, 161, 119	1802		++
	m/z: 157, 143, 185, 270	1857	+	
*	Bis(2-methylpropyl)phthalat*	1866	++	++
	Heptadecen-2-on	1874		++
	Nonadecan	1896		++
*	Di-2-pyridinylethanedion*	1906		++
*	Dibutylphthalat*	1961	+	++
	m/z: 239, 183, 253, 268	1988	+	
	Eicosane	1995		++
aq	Octasulfur	2025		++++

[a)]Peak in chromatogram, [b)]Abundance relative to the largest peak in the total ion chromatogram: + 0 – 0.5%; ++ 0.5 – 10%; +++ 10 – 30%; ++++ 30 – 100%, [c)]not determined, *) artifact, grey shading – also identified in medium.

Figure S2C Total ion chromatogram and compounds identified of *B. subtilis* headspace. Largest peaks are indicated with a letter which corresponds with the list of compounds below.

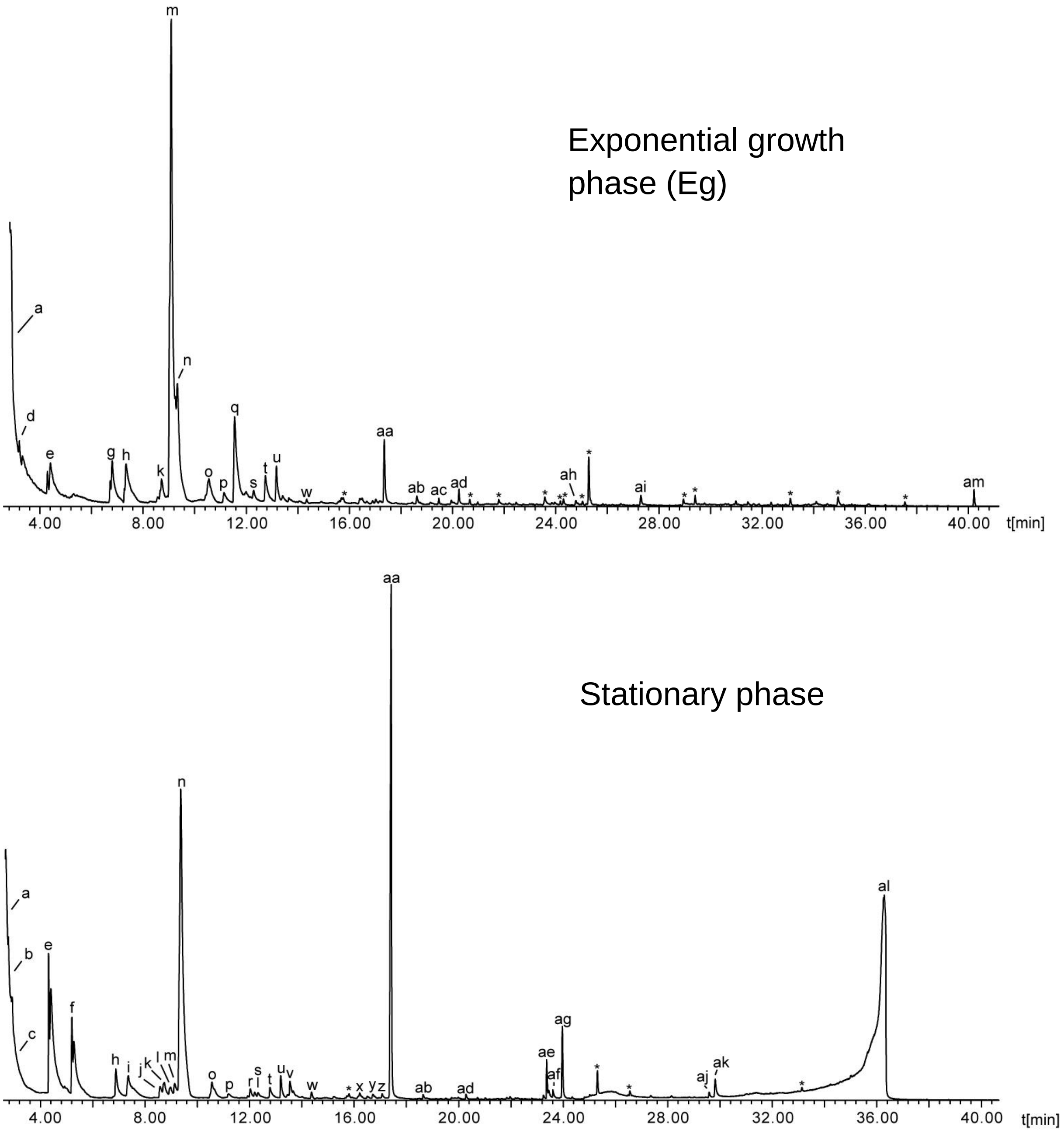

GC[a)]	Compound	Retention index[b)]	Intensity A	Intensity B
a	Acetoin	n.e.[c)]	++	++
b	3-Methyl-1-butanol	n.e.		++
c	Dimethyldisulfide	n.e.		++
d	Toluol	n.e.	++++	
e	Butylacetate	826	+++	++++
f	m/z: 88, 43, 55	854		+++
g	Cyclohexanone	901	+++	

h	3-(Acetyloxy)-2-butanone	903		++
i	2,5-Dimethylpyrazine	915	+++	++
j	2-Hydroxy-3-heptanon or isomere	947		++
k	3-Hydroxy-2-heptanon or isomere	952	++	++
l	Butylisobutyrate	958		++
m	Benzaldehyde	963	++++	++
n	Dimethyltrisulfide	968	++++	++++
o	Trimethylpyrazine	999	++	++
p	2-Acetylthiazol	1016	++	++
q	2-Ethyl-1-hexanol*	1029	+++	
	Ethyl-2-isopropyl-2-butenoate	1042	++	
r	Butyl-2-methylbutanoate	1042		++
s	Pyrazine, M: 136	1050	++	++
t	Acetophenone	1063	++	++
u	2-Ethyl-3,6-dimethylpyrazine	1075	++	++
	Pyrazine, M: 136	1082	+	++
v	2-Methoxyphenol	1087		++
	Ethylbenezenediol	1090	+	++
w	m/z: 43, 123, 101, 138	1111	+	++
	m/z: 132, 67, 99, 53, 39	1139		+
	m/z: 97, 59, 140	1150	+	
	Pyrazine, M: 150	1151	+	+
	2,6-Di(1-Methylethyl)pyrazine	1153	+	+
*	Silanoxane	1156	+	+
x	Unknown, MW: 150	1170		++
	Naphthalene	1176	+	
y	Tetrathiolane	1187		+
z	Dimethyl-(2-methylpropyl)-Pyrazine M=164	1196		++
aa	Dimethyltetrasulfide	1208	++	++++
ab	2-Butyl-3,6-dimethylpyrazine	1253	++	++
ac	Isobornylacetate	1285	++	+
	2-Undecanone	1292		+
	Pyrazine	1302	+	
ad	2,5-Dimethyl-3-(3-methylbutyl)-pyrazine	1313	++	++
*	Siloxane	1329	+	+
	m/z: 138, 95, 83, 193, 57	1340	+	+
*	Unknown	1371	+	+
	m/z: 91, 71, 99, 117, 55	1379		+
	Pyrazine	1388		+
	m/z: 113, 192, 45, 136,	1390		+
	m/z: 71, 117, 43	1428		++
ae	Isobutyl-2-phenylacetate	1432		++
af	Pentathiane	1437		++
*	Unknown	1442	++	++
ag	Dimethylpentasulfide	1455		++
*	Unknown	1465	++	
*	Cyclohexadien-1,4-dion-Derivate	1470	+	+
ah	5-Methyl-2-phenyl-2-hexenal	1488	+	
	Pentadecane	1498		+
*	Cyclosiloxane	1499	+	

*	2,4-Bis(1,1-dimethylethyl)-phenol	1509	++	++
ai	Hexadecane	1596	++	
*	Cyclosiloxane	1669	+	
aj	2-Pentadecanone	1695		++
ak	Hexathiepane	1707		++
	14-Methyl-2-pentadecanone	1760	+	
*	Unknown	1763	+	
	13-Methyl-2-pentadecanone	1771	+	
	Methyl-13-methyltetradecanoat	1786	+	
*	Unknown	1829	+	
*	Bis(2-methylpropyl)phthalate	1866	++	+
*	Dibutylphthalate	1961	++	
	m/z: 239, 183, 253, 268	1988	+	
al	Octasulfur	2025		++++
	m/z: 237, 195, 241, 221, 252	2058	+	
	Dodecanoic acid ethylester	2099	+	
am	m/z: 149, 91, 121, 79	2253	++	

[a)]Peak in chromatogram, [b)]Abundance relative to the largest peak in the total ion chromatogram: + 0 – 0.5%; ++ 0.5 – 10%; +++ 10 – 30%; ++++ 30 – 100%, [c)]not determined, *) artifact, grey shading – also identified in medium.

Figure S2D Total ion chromatogram of *S. epidermidis* headspace and compounds identified

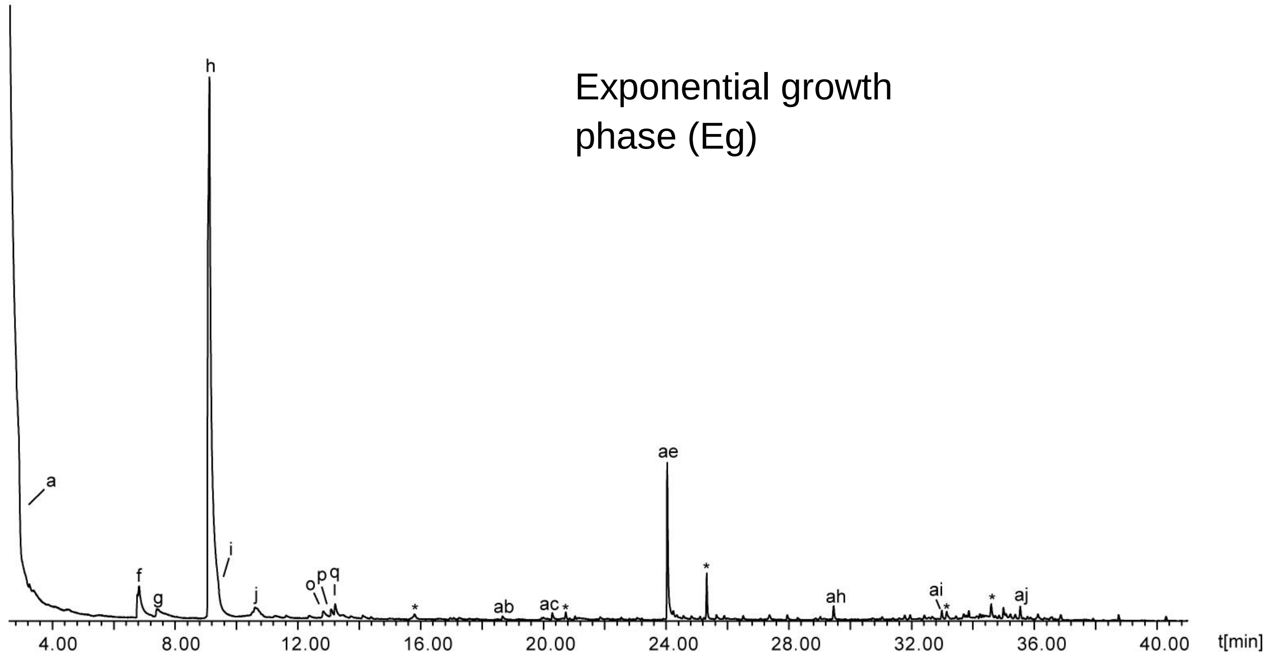

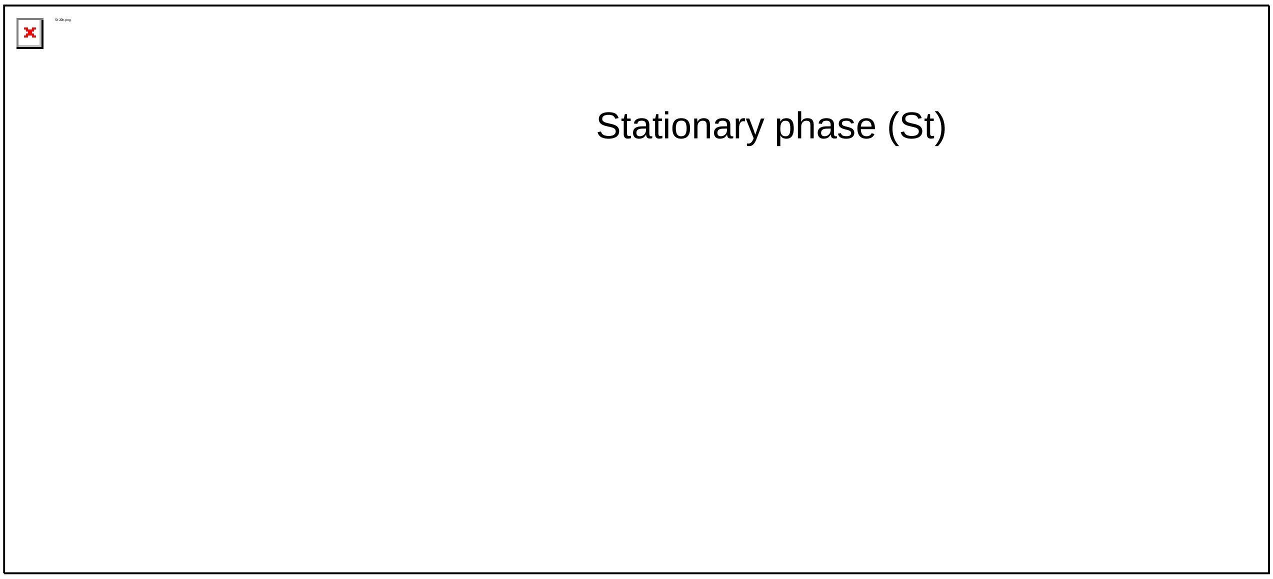

GC[a)]	Compound[b)]	Retention index[c)]	Intensity A	B
a	1-Butanol	n.e.[c)]	+++	
b	Acetoin	n.e.		+++
c	3-Methyl-1-butanol	n.e.		+++
d	Dimethyldisulfide	n.e.		+++
e	Butylacetate	826		++++
f	Cyclohexanone	901	++	++
g	2,5-Dimethylpyrazine	915	++	+++
h	Benzaldehyde	963	++++	++++
i	Dimethyltrisulfide	968	+++	++++

	Butylbutyrate	997	+	++
j	Trimethylpyrazine	999	+	++
k	2-Acetylthiazol	1016		++
l	2-Ethyl-1-hexanol*	1029		++
	Ethyl 2-isopropyl-2-butenoate	1042		+
m	Butyl-2-methylbutanoate	1042		++
n	Pyrazine, M: 136	1050	+	++
	S-Methyl-methanthiosulphonate	1062		+
o	Acetophenone	1063	++	++
p	5-Nonanone	1073	++	+
q	2-Ethyl-3,6-dimethylpyrazine	1075	++	++
r	Pyrazine, M: 136	1082		++
s	Ethylbenezenediol	1090	+	++
	Nonanal	1104	+	+
t	m/z: 43, 123, 101, 138	1111	+	++
u	Phenylacetonitril	1138		+
v	Pyrazine, M: 150	1151		+
w	2,6-Di(1-Methylethyl)pyrazine	1153		++
x	8-Methyl-2-nonanone	1155		++
*	Silanoxan	1156	+	++
	7-Methyl-2-nonanone	1160		+
	Pyrazine, M=164	1193	+	
y	Dimethyl-(2-methylpropyl)-Pyrazine	1196	+	++
z	Decanal	1201		++
aa	Dimethyltetrasulfide	1208		+++
ab	2-Butyl-3,6-dimethylpyrazine	1253	+	++
	α-Ethylidenebenzeneacetaldehyd e	1272		+
	2-Undecanone	1292		+
	Indol	1294		+
	m/z: 57, 143, 87, 69, 159	1301	+	
	Pyrazine	1302	+	+
	m/z: 57, 143, 87, 159, 69	1303		+
ac	2,5-Dimethyl-3-(3-methylbutyl)-pyrazine	1313	+	++
	m/z: 138, 95, 83, 193, 57	1340	++	
	10-Methyl-2-undecanone	1359		+
	9-Methyl-2-undecanone	1363		+
	Pyrazine	1388		+
	Tetradecane	1397	+	+
	Sesquiterpen: Cadina-1,4-dien	1420		+
ad	Dimethylpentasulfide	1455		++
ae	6-Pentyl-2H-Pyran-2-on*	1456		++
	Cyclohexadien-1,4-dion-derivate*	1470	+	+
	5-Methyl-2-phenyl-2-hexenal	1488	+	++
	2-Tridecanone	1496		+
*	Cyclosiloxane	1499	+	+
	2,4-Bis(1,1-dimethylethyl)-phenol	1509	++	++
	Dodecanoic acidmethylester	1530	+	++
	m/z: 159, 202, 129, 131	1535	+	
	12-Methyl-2-tridecanone	1557		+
	m/z: 193, 55, 222, 95, 43	1562	+	

af	m/z: 69, 93, 41, 107, 71	1563		+
	11-Methyl-2-tridecanone	1567		+
ag	m/z: 107, 163, 56, 79, 41	1586		+
	Hexadecane	1596		++
*	Unknown	1598	+	
	Cyclosiloxane	1669	+	
ah	m/z: 109, 135, 82, 123, 93	1692		+
	2-Pentadecanone	1695		+
	Hexathiepane	1707		+
	14-Methyl-2-pentadecanone	1760		+
	m/z: 253, 268, 185, 171, 85	1800	+	
	m/z: 229, 145, 159, 161,119	1802	+	+
ai	m/z: 143, 157, 270, 185, 200	1861	++	+
*	Bis(2-methylpropyl)phthalate*	1866	+	++
	m/z: 119, 105, 272, 204, 91	1884		+
	m/z: 145, 160, 270, 95, 106	1905	++	+
	m/z: 160, 145, 270, 95, 105	1943	++	++
	m/z: 243, 91, 258, 147, 133	1950		+
	m/z: 243, 91, 119, 161, 105	1968		+
	m/z: 256, 157, 131, 143, 118	1975		+
aj	m/z: 239, 183, 253, 268	1988	++	
	2-Octadecanone	2004		+
ak	Octasulfur	2025		++
	m/z: 197, 282, 254, 239, 141	2121	+	++
	Diterpene	2168	++	++
	m/z: 231, 246, 108, 121, 81	2211		+
	m/z: 121, 91, 79, 105, 93	2257	+	
al	m/z: 149, 91, 121, 79, 243	2257		++

a) Peak in chromatogram, b) Artefacts, c)Abundance relative to the largest peak in the total ion chromatogram: + 0 – 0.5%; ++ 0.5 – 10%; +++ 10 – 30%; ++++ 30 – 100%, d) not determined

Figure S2E Total ion chromatogram of *B. epidermidis* headspace and compounds identified

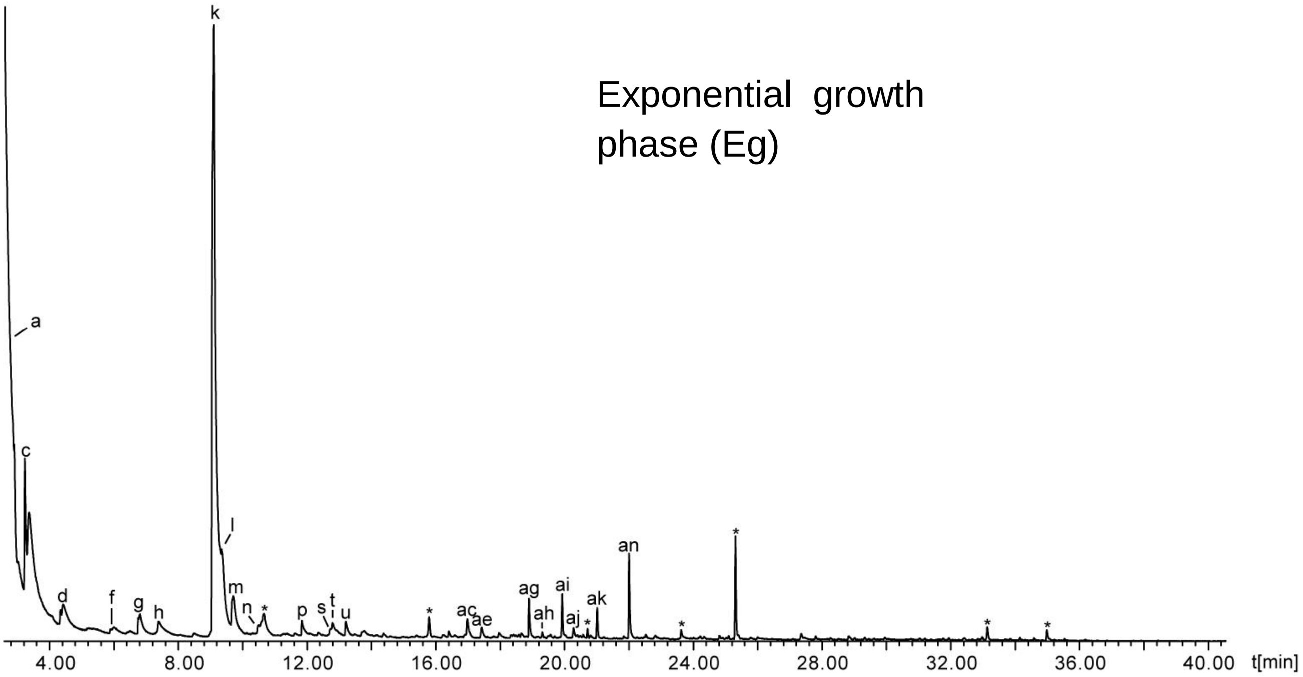

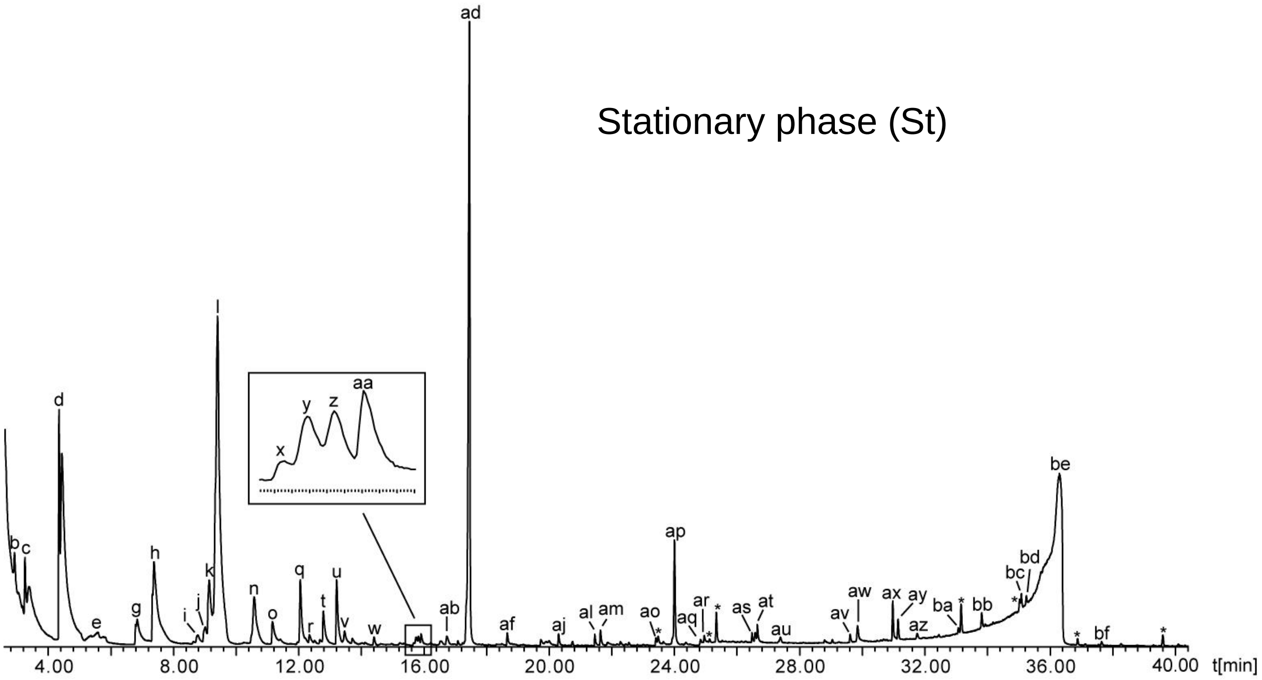

GC[a)]	Compound[b)]	Retention index[c)]	Intensity A	Intensity B
a	1-Butanol	n.e.[c)]	++	
b	Dimethyldisulfide	n.e.		++
c	Toluol	n.e.	+++	+++
d	Butylacetate	826	++	++++
e	3-Methylbutanoic acid	863		++
f	Xylol	872	+	

g	Cyclohexanone	901	++	++
h	2,5-Dimethylpyrazine	915	++	+++
i	3-Hydroxy-2-heptanon or isomere	952		++
j	Butylisobutyrate	958		++
k	Benzaldehyde	963	++++	++
l	Dimethyltrisulfide	968	+++	++++
m	1,2-Dithiolane	978	++	
n	Trimethylpyrazine	999	++	++
*	Cyclosiloxan	1003	++	
o	2-Acetylthiazole	1016		++
p	2,3-Dimethyl-2-cyclopenten-1-one	1037	++	
q	Butyl-2-methylbutanoate	1042		++
r	Pyrazine, M: 136	1050	+	++
s	S-Methyl-methanthiosulphonate	1062	++	
t	Acetophenone	1063	++	++
u	2-Ethyl-3,6-dimethylpyrazine	1075	++	++
v	Pyrazine, M: 136	1082		++
	Ethylbenezenediol	1090		+
	m/z: 43, 123, 101, 138	1111	+	++
x	Pyrazine, M: 150	1151		+
y	2,6-Di(1-Methylethyl)pyrazine	1153		++
z	Pyrazine, M: 150	1155		+
*	Silanoxane	1156	+	
aa	7-Methyl-2-nonanone	1160		+
ab	Tetrathiolane	1187		++
ac	Sulfur compound	1195	++	
	Dimethyl-(2-methylpropyl)-pyrazine M=164	1196		+
ad	Dimethyltetrasulfide	1208		++++
ae	Indan Derivate	1210	++	
af	2-Butyl-3,6-dimethylpyrazine	1253	+	++
ag	1-Chlorodecane	1263	++	
ah	3-Methyldodecane	1279	+	
	2-Undecanone	1292		+
	Indol	1294		+
ai	m/z: 57, 143, 87, 69, 159	1301	++	
	Pyrazine	1302		+
aj	2,5-Dimethyl-3-(3-methylbutyl)-pyrazine	1313	++	++
ak	m/z: 138, 95, 83, 193, 57	1340	++	
al	10-Methyl-2-undecanone	1359		++
am	9-Methyl-2-undecanone	1363		++
an	m/z: 91, 71, 99, 117, 55	1379	++	
	Pyrazine	1388		+
	m/z: 113, 192, 45, 136	1390		+
	Tetradecane	1397	+	+
	Isobutyl-2-phenylacetate	1432		+
ao	Pentathiane	1437		++
*	Unknown	1442	++	
ap	Dimethylpentasulfide	1455		++
	Hexasulfur	1487	+	+
aq	5-Methyl-2-phenyl-2-hexenal	1488	+	+

ar	2-Tridecanone	1496		+
*	2,4-Bis(1,1-dimethylethyl)-phenol	1509	++	++
as	12-Methyl-2-tridecanone	1557		++
at	11-Methyl-2-tridecanone	1567		++
au	Hexadecane	1596		+
	m/z: 71, 43, 139, 224, 97	1609	+	
	13-Methyl-2-tetradecanone	1662		+
av	2-Pentadecanone	1695		++
aw	Hexathiepane	1707		++
ax	14-Methyl-2-pentadecanone	1760		++
ay	13-Methyl-2-pentadecanone	1771		++
az	2-Hexadecanone	1800		++
ba	15-Methyl-2-hexadecanone	1865		++
*	Bis(2-methylpropyl)phthalat	1866	++	++
bb	2-Heptadecanone	1902		++
	m/z: 160, 145, 270, 95, 105	1943	+	
*	Dibutylphthalat	1961	++	++
bc	16-Methyl-2-heptadecanone	1968		++
bd	15-Methyl-2-heptadecanone	1976		++
	m/z: 239, 183, 253, 268	1988	+	
	2-Octadecanone	2004		+
be	Octasulfur	2025		++++
bf	2-Nonadecanone	2105		+
*	Cyclosiloxan	2217		+

a) Peak in chromatogram, b) Artefacts, c)Abundance relative to the largest peak in the total ion chromatogram: + 0 – 0.5%; ++ 0.5 – 10%; +++ 10 – 30%; ++++ 30 – 100%, d) not determined

Figure S2F Total ion chromatogram of *P. aeruginosa* headspace and compounds identified

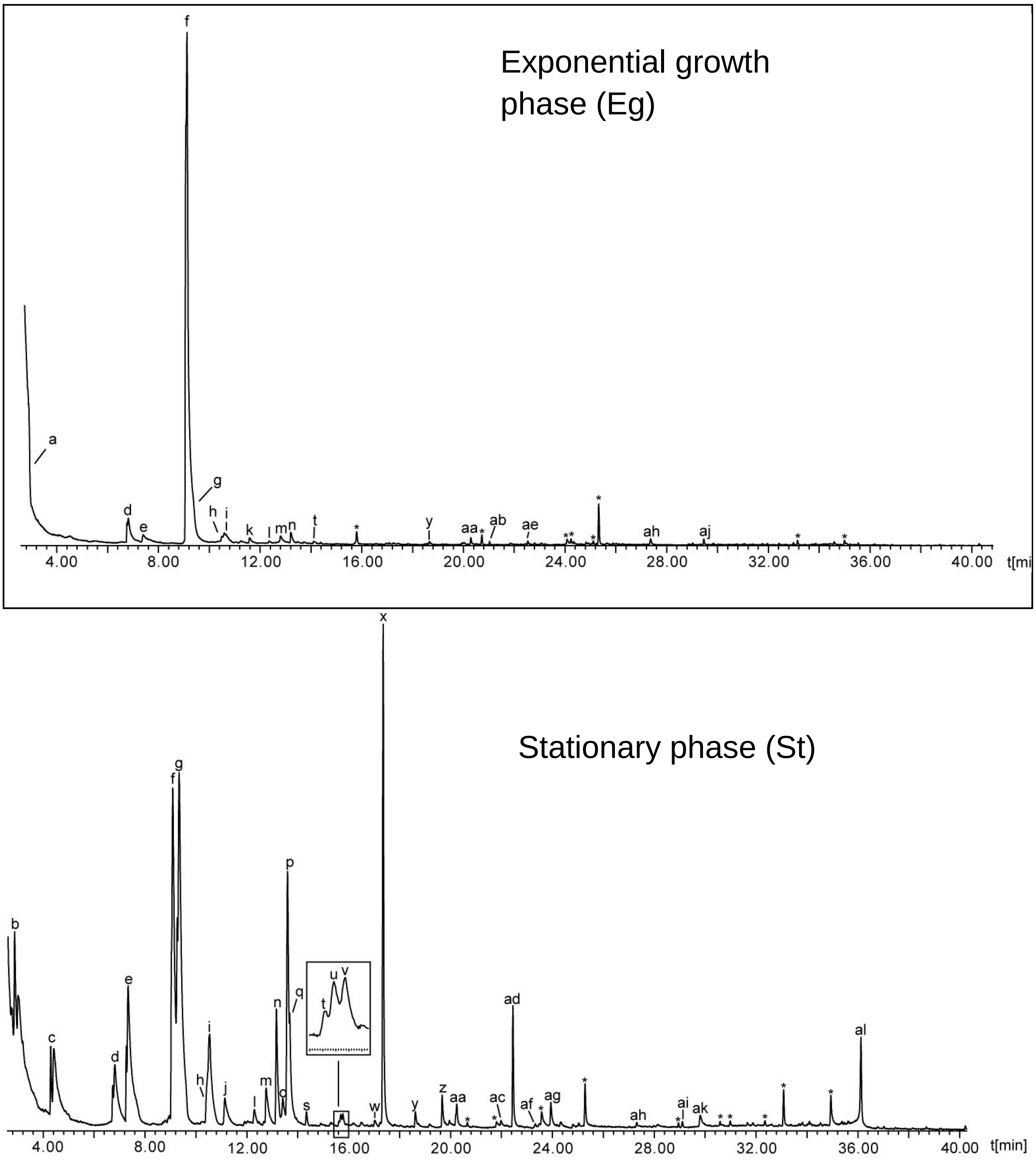

GC[a)]	Compound[b)]	Retention index[c)]	Intensity A	Intensity B
a	1-Butanol	n.e.[c)]	++	
b	Dimethyldisulfide	n.e.		+++
c	Butylacetate	826		+++
d	Cyclohexanone	901	++	+++
e	2,5-Dimethylpyrazine	915	++	++++
	Butylisobutyrate	958		+
f	Benzaldehyde	963	++++	++++

g	Dimethyltrisulfide	968	+++	++++
h	Butylbutyrate	997	++	++
i	Trimethylpyrazine	999	++	++++
j	2-Acetylthiazole	1016		++
k	2-Ethyl-1-hexanol	1029	++	
l	Pyrazine, M: 136	1050	++	++
m	Acetophenone	1063	++	++
n	2-Ethyl-3,6-dimethylpyrazine	1075	++	+++
o	Pyrazine, M: 136	1082	+	++
p	1-Undecene	1088		++++
q	Methylbenzoate	1091		+++
r	Nonanal	1104	+	
s	m/z: 43, 123, 101, 138	1111		++
t	Pyrazine, M: 150	1151		+
u	2,6-Di(1-Methylethyl)pyrazine	1153		+
v	Pyrazine, M: 150	1155		+
*	Silanoxan	1156	++	
w	Pyrazine, M=164	1193	+	
	Dimethyl-(2-methylpropyl)-pyrazin M=164	1196		+
x	Decanal	1201	+	
	Dimethyltetrasulfide	1208		++++
	2-Butyl-3,6-dimethylpyrazine	1253	+	++
z	2-Undecanone	1292		++
	m/z: 57, 143, 87, 69, 159	1301	+	
	Pyrazine	1302	+	
aa	2,5-Dimethyl-3-(3-methylbutyl)-pyrazine	1313	++	++
	m/z: 69, 43, 85, 111,154	1321	+	
ab	m/z: 138, 95, 83, 193, 57	1340	+	
ac	Nerylmethylthioether	1379		+
ad	Geranylmethylthioether	1396		+++
ae	Tetradecan	1397	+	
af	Diphenylmethane	1430		+
ag	Dimethylpentasulfid	1455		++
*	Cyclohexadien-1,4-dion-Derivat	1470	+	+
	5-Methyl-2-phenyl-2-hexenal	1488	+	
*	Cyclosiloxane	1499	+	
*	2,4-Bis(1,1-dimethylethyl)-phenol	1509	++	++
ah	Hexadecane	1596	++	+
ai	Hydrocarbon	1677		+
aj	m/z: 109, 82, 135, 121, 150	1692		+
ak	Hexathiepan	1707		+
	m/z: 229, 145, 159, 161,119	1802	+	
	m/z: 157, 143, 185, 270	1857	+	
*	Bis(2-methylpropyl)phthalat	1866	++	
	m/z: 145, 160, 270, 95, 106	1905	+	
	m/z: 160, 145, 270, 95, 105	1943	+	
*	Dibutylphthalat	1961	+	++
al	Octasulfur	2025	+	+++

a) Peak in chromatogram, b) Artefacts, c)Abundance relative to the largest peak in the total ion chromatogram: + 0 – 0.5%; ++ 0.5 – 10%; +++ 10 – 30%; ++++ 30 – 100%, d) not determined
