# Supplementary material for: Differential Attraction of Malaria Mosquitoes to Volatile Blends Produced by Human Skin Bacteria
Source: PLoS One. 2010 Dec 30;5(12):e15829. doi: 10.1371/journal.pone.0015829 (PMC3012726; doi:10.1371/journal.pone.0015829)
Supplement: Table S1 — Bacterial species used for experiments and headspace analyses. (DOC) [file pone.0015829.s005.doc]

**Table S1**. **Bacterial species used for experiments and headspace analyses.**

| **Species** | **Origin** |
| --- | --- |
| *Staphylococcus epidermidis* | DSMZ, Braunschweig, Germany, Nr. 20044 |
| *Corynebacterium minutissimum* | DSMZ, Braunschweig, Germany, Nr. 20651 |
| *Brevibacterium epidermidis* | DSMZ, Braunschweig, Germany, Nr. 20660 |
| *Pseudomonas aeruginosa* | Laboratory of Microbiology, Wageningen University, The Netherlands, Isolate P8, Delft, 1957 |
| *Bacillus subtilis* | Laboratory of Microbiology, Wageningen University, The Netherlands, Isolate B28, Marburg strain, J.W. Woldendorp. Delft, 1961 |
