# Supplementary material for: Differential Attraction of Malaria Mosquitoes to Volatile Blends Produced by Human Skin Bacteria
Source: PLoS One. 2010 Dec 30;5(12):e15829. doi: 10.1371/journal.pone.0015829 (PMC3012726; doi:10.1371/journal.pone.0015829)
Supplement: Table S2 — Relative abundance of compounds that were potentially attractive or repellent to A. gambiae. (DOC) [file pone.0015829.s006.doc]

**Table S2. Relative abundance of compounds that were potentially attractive or repellent to *A. gambiae*.**

A

| **Compounds** | **RI** | ***Bac*** | ***Bac*** | ***Bre*** | ***Bre*** | ***Cor*** | ***Cor*** | ***Sta*** | ***Sta*** | ***Pse*** | ***Pse*** |
| --- | --- | --- | --- | --- | --- | --- | --- | --- | --- | --- | --- |
|  |  | **Eg** | **St** | **Eg** | **St** | **Eg** | **St** | **Eg** | **St** | **Eg** | **St** |
| Dimethyldisulfide | n.d. ^1^ |  | ++ |  | ++ |  |  |  | +++ |  | +++ |
| Butyl acetate | 826 | +++ | ++++ | ++ | ++++ |  | ++++ |  | ++++ |  | +++ |
| Butyl isobutyrate | 958 |  | ++ |  | ++ |  | ++ |  |  |  | + |
| Butyl 2-methylbutanoate | 1042 |  | ++ |  | ++ |  | +++ |  | ++ |  |  |
| Dimethyltetrasulfide^2^ | 1208 | ++ | ++++ |  | ++++ |  | ++++ |  | +++ |  | ++++ |
| Pentathiane^2^ | 1437 |  | ++ |  | ++ |  | ++ |  |  |  |  |
| Dimethylpentasulfide^2^ | 1455 |  | ++ |  | ++ |  | ++ |  | ++ |  | ++ |
| 2-Pentadecanone | 1695 |  | ++ |  | ++ |  | ++ |  | + |  |  |
| Hexathiepane^2^ | 1707 |  | ++ |  | ++ |  | ++ |  | + |  | + |
| Octasulfur^2^ | 2025 |  | ++++ |  | ++++ |  | ++++ |  | ++ | + | +++ |

B

| **Compounds** | **RI** | ***Bac*** | ***Bre*** | ***Cor*** | ***Sta*** | ***Pse*** |
| --- | --- | --- | --- | --- | --- | --- |
| Butyl butyrate | 997 |  |  |  | ++ | ++ |
| Butyl 2-methylbutanoate | 1042 | ++ | ++ | +++ | ++ |  |
| Pentathiane^2^ | 1437 | ++ | ++ | ++ |  |  |
| 2-Pentadecanone | 1695 | ++ | ++ | ++ | + |  |

Compounds were selected from the volatiles detected in the headspace of broths of five different bacterial species. The selection criteria are described in the Material and Methods section. **A**: Exponential growth phase (Eg) compared with stationary phase (St) of *B. subtilis (Bac), Brev. epidermidis (Bre), C. minutissimum (Cor)* and *S. epidermidis (Sta)*. The abundance of *P. aeruginosa (Pse)* is given as a reference; **B:** Stationary phase of *P. aeruginosa (Pse)* compared with the stationary phase of the other four bacterial species. Abundance based on GC-MS analysis: + 0 – 0.5%; ++ 0.5 – 10%; +++ 10 – 30%; ++++ 30 – 100%, relative to the largest peak in the total ion chromatogram. For the full list of compounds identified and chromatograms, see supplementary material Figure S2. ^1^not determined, ^2^not commercially available.
