# Supplementary material for: Differential Attraction of Malaria Mosquitoes to Volatile Blends Produced by Human Skin Bacteria
Source: PLoS One. 2010 Dec 30;5(12):e15829. doi: 10.1371/journal.pone.0015829 (PMC3012726; doi:10.1371/journal.pone.0015829)
Supplement: Table S3 — Medium ingredients used for initial growth of each bacterial species (DSMZ, Germany). (DOC) [file pone.0015829.s007.doc]

**Table S3**. **Medium ingredients used for initial growth of each bacterial species (DSMZ, Germany).**

| **Bacterial species** | **Ingredients** | **Amount** | **Supplier** |
| --- | --- | --- | --- |
| *B. subtilis, P. aeruginosa* | Proteose peptone | 5.0 g | Fluka |
|  | Meat extract | 3.0 g | Fluka |
|  | Distilled water | 1000 ml |  |
|  |  |  |  |
| *Brev. epidermidis, S. epidermidis* | Pancreatic digest of casein (Peptone C) | 10.0 g | Difco |
|  | Yeast extract | 5.0 g | Difco |
|  | Glucose | 5.0 g | Merck |
|  | Sodium chloride (≥ 99.5%) | 5.0 g | Merck |
|  | Distilled water | 1000 ml |  |
|  |  |  |  |
| *C. minutissimum* | Infusion from heart muscle | 18.5 g | Fluka |
|  | Glucose | 5.0 g | Merck |
|  | Distilled water | 1000 ml |  |
