# Supplementary material for: Differential Attraction of Malaria Mosquitoes to Volatile Blends Produced by Human Skin Bacteria
Source: PLoS One. 2010 Dec 30;5(12):e15829. doi: 10.1371/journal.pone.0015829 (PMC3012726; doi:10.1371/journal.pone.0015829)
Supplement: Table S4 — Ingredients of standard liquid medium used for growth of the five bacterial species. (DOC) [file pone.0015829.s008.doc]

**Table S4. Ingredients of standard liquid medium used for growth of the five bacterial species.**

| **Ingredient** | **Amount** | **Supplier** |
| --- | --- | --- |
| Infusion from heart muscle | 2.0 g | Fluka |
| Pancreatic digest of casein (Peptone C) | 13.0 g | Difco |
| Yeast extract | 5.0 g | Difco |
| Sodium chloride (≥ 99.5%) | 5.0 g | Merck |
| Distilled water | 1000 ml |  |
