# Supplementary material for: Differential Attraction of Malaria Mosquitoes to Volatile Blends Produced by Human Skin Bacteria
Source: PLoS One. 2010 Dec 30;5(12):e15829. doi: 10.1371/journal.pone.0015829 (PMC3012726; doi:10.1371/journal.pone.0015829)
Supplement: Table S5 — Correlation between the number of CFUs in liquid medium and the extinction coefficient. (DOC) [file pone.0015829.s009.doc]

**Table S5**. **Correlation between the number of CFUs in liquid medium and the extinction coefficient.**

| **Bacterial species** | **Linear equation** | **R^2^ (%)** |
| --- | --- | --- |
| *B. subtilis* | Y = 1.452E-09X | 96.0 |
| *Brev. epidermidis* | Y = 2.349E-09X | 99.2 |
| *C. minutissimum* | Y = 6.035E-09X | 98.2 |
| *P. aeruginosa* | Y = 9.883E-10X | 98.6 |
| *S. epidermidis* | Y = 3.877E-09X | 91.8 |

Extinction was measured in a spectrophotometer at 620 nm. Y = Extinction coefficient. X = Number of CFUs. R^2^ = coefficient of determination.
