# Supplementary material for: Differential Attraction of Malaria Mosquitoes to Volatile Blends Produced by Human Skin Bacteria
Source: PLoS One. 2010 Dec 30;5(12):e15829. doi: 10.1371/journal.pone.0015829 (PMC3012726; doi:10.1371/journal.pone.0015829)
Supplement: Table S6 — Exponential growth rate and fitted equation of the bacterial species in liquid medium. (DOC) [file pone.0015829.s010.doc]

**Table S6. Exponential growth rate and fitted equation of the bacterial species in liquid medium.**

| **Bacterial species** | **Growth optimum (h)** | **Logistic growth equation** | **R^2^ (%)** |
| --- | --- | --- | --- |
| *B. subtilis* | 15.3 | Y = -0.044 + 4.840/(1 + EXP(-0.374*(T – 15.27))) | 96.1 |
| *Brev. epidermidis* | 8.7 | Y = -0.0794 + 11.262/(1 + EXP(-0.341*(T – 8.717))) | 97.8 |
| *C. minutissimum* | 16.5 | Y = -0.979 + 11.406/(1 + EXP(-0.1799*(T – 16.518))) | 98.3 |
| *P. aeruginosa* | 13.0 | Y = -0.092 + 5.151/(1 + EXP(-0.320*(T – 13.02))) | 89.7 |
| *S. epidermidis* | 10.9 | Y = -0.223 + 8.310/(1 + EXP(-0.470*(T – 10.900))) | 94.7 |

Y = Extinction coefficient. T = Time (hours). R^2^ = coefficient of determination.
